# Human pluripotent stem cell-derived cardiomyocytes as a target platform for paracrine protection by cardiac mesenchymal stromal cells

**DOI:** 10.1038/s41598-020-69495-w

**Published:** 2020-08-03

**Authors:** Chrystalla Constantinou, Antonio M. A. Miranda, Patricia Chaves, Mohamed Bellahcene, Andrea Massaia, Kevin Cheng, Sara Samari, Stephen M. Rothery, Anita M. Chandler, Richard P. Schwarz, Sian E. Harding, Prakash Punjabi, Michael D. Schneider, Michela Noseda

**Affiliations:** 1 National Heart and Lung Institute, Imperial College London, Du Cane Road, London, W12 0NN UK; 2 British Heart Foundation Centre for Research Excellence and Centre for Regenerative Medicine, Imperial College London, London, W12 0NN UK; 3 Kardia Therapeutics, Houston, TX 77030 USA; 4 Hammersmith Hospital, Imperial College Healthcare NHS Trust, London, W12 0HS UK; 5 Present Address: Department of Bioengineering, BioScience Research Collaborative, Rice University, Houston, TX 77005 USA; 6 Present Address: CV Ventures, LLC, Blue Bell, PA 19422 USA

**Keywords:** Cardiac regeneration, Cardiovascular diseases, Induced pluripotent stem cells

## Abstract

Ischemic heart disease remains the foremost cause of death globally, with survivors at risk for subsequent heart failure. Paradoxically, cell therapies to offset cardiomyocyte loss after ischemic injury improve long-term cardiac function despite a lack of durable engraftment. An evolving consensus, inferred preponderantly from non-human models, is that transplanted cells benefit the heart via early paracrine signals. Here, we tested the impact of paracrine signals on human cardiomyocytes, using human pluripotent stem cell-derived cardiomyocytes (hPSC-CMs) as the target of mouse and human cardiac mesenchymal stromal cells (cMSC) with progenitor-like features. In co-culture and conditioned medium studies, cMSCs markedly inhibited human cardiomyocyte death. Little or no protection was conferred by mouse tail tip or human skin fibroblasts. Consistent with the results of transcriptomic profiling, functional analyses showed that the cMSC secretome suppressed apoptosis and preserved cardiac mitochondrial transmembrane potential. Protection was independent of exosomes under the conditions tested. In mice, injecting cMSC-conditioned media into the infarct border zone reduced apoptotic cardiomyocytes > 70% locally. Thus, hPSC-CMs provide an auspicious, relevant human platform to investigate extracellular signals for cardiac muscle survival, substantiating human cardioprotection by cMSCs, and suggesting the cMSC secretome or its components as potential cell-free therapeutic products.

## Introduction

The paramount public health burden of ischemic heart disease^1^ and the dearth of restorative growth in adult mammalian myocardium have, together, given focus to maintaining cardiomyocyte number as a therapeutic target^2,3^. If successful, this would transform the scope of treatment for ischemic heart disease, beyond merely reperfusion to restore coronary flow. Proposed approaches include diverse cell therapies aimed at the generation of new cardiac muscle, by grafting putative precursors such as bone marrow stem cells, heart-derived cardiac progenitor/stem cells, cardiac mesenchymal cells, cardiosphere-derived cells, and established cardiomyocytes made from pluripotent stem cells^2,3^. However, the persistence of grafted cells in recipient hearts is brief, at least in the pre-clinical setting where it can be tracked conclusively, leading to a persuasive two-fold paradigm shift^4–13^. First, therapeutic grafting to supply de novo contractile myocytes themselves is now understood to require alternatives beyond simplistic injections of naked cells, an aspect ripe for biomaterials and tissue engineering strategies^14^. Second, more fundamentally, alternative mechanisms must be sought to reconcile the transience of grafted cells with the long-term benefits to cardiac structure and function. Consequently, paracrine signals early after grafting have been viewed as the most cogent explanation, with reported benefits including infarct regression, cardiomyocyte proliferation, stem cell recruitment, immunomodulation, attenuation of fibrosis, and enhanced contractile function^4–13^. These findings have overt translational significance, yet have surprisingly little validation to date using a human cardiac model as target.

Based on animal studies, one proposed benefit independent of any long-term engraftment is cardioprotection, i.e., the direct enhancement of cardiomyocytes’ survival^7,15,16^. Cardioprotection is the ambition of many human trials, which have routinely failed to demonstrate efficacy^16,17^. One limitation, amongst others, is the almost exclusive reliance on animal models alone^18^, and the lack of a concerted approach to establish human preclinical benefits. This limitation is especially striking, when compared to oncology, which has long relied on human cancer cell lines as pivotal preclinical models for target validation, mechanistic studies, and drug development, as a reliable and instructive platform propelling innovation^19^. By direct analogy, human pluripotent stem-cell derived cardiomyocytes (hPSC-CMs) can now provide unprecedentedly routine, scalable, transformative access to human cardiac biology for experimental therapeutics^20–22^, including drug discovery to enhance cardiomyocyte survival^23,24^.

Previously, we described a subpopulation of cardiac stromal cells that provide protection from acute ischemic injury after intramyocardial injection^5^. This subset of cardiac stromal cells is enriched for two surface markers—Sca1 and PDGFRα—and exhibits the side population (SP) dye-efflux phenotype typical of stem cells from bone marrow and diverse solid other organs^25,26^. These cells comprise the most highly clonogenic population found to date in adult mammalian hearts, reduce infarct size after grafting, demonstrate cardiogenic potential (in the very small number of persisting cells), and most strongly resemble the cardiac colony-forming unit-fibroblast and related cardiac mesenchymal cells^4,5,27^. Whether such cells contribute to cardiac myocyte formation, natively, is disputed on the basis of fate-mapping studies^28–31^. To be conservative, we therefore refer to this subpopulation as cardiac mesenchymal stromal cells (cMSC). Notably, the mechanisms mediating cMSCs' cardioprotective effects remain unclear, and a paracrine hypothesis is the most plausible given these cells’ poor retention in the myocardium, a limitation also found with the vast majority of cell therapies when injected as a simple cell suspension^4–13^.

Here, we developed a system to test the putative protective paracrine effect of cMSC explicitly in human cardiomyocytes. We report proof-of-principle studies in two independent hPSC-CM lines, demonstrating potent protection from lethal oxidative stress by paracrine signals from mouse and human adult cardiac stromal cells that share similar cardiac progenitor-like molecular features. Moreover, human cardiomyocyte protection was generalizable, being shown, further, in the context of two clinically relevant cardiotoxic anti-cancer drugs. We also demonstrate the potential for benefits in vivo, confirming that intramyocardial injection of cMSC-conditioned media can suppress cardiomyocyte apoptosis locally in a mouse model of myocardial infarction (MI). These results establish directly that paracrine factors from mouse and human cMSCs can benefit human cardiomyocyte survival and give credence to exploring cMSC-conditioned medium or its components as cell-free therapeutic products.

## Results

### Suppression of cell death by mouse cMSC in human cardiomyocytes derived from pluripotent stem cells

**Figure 1:**
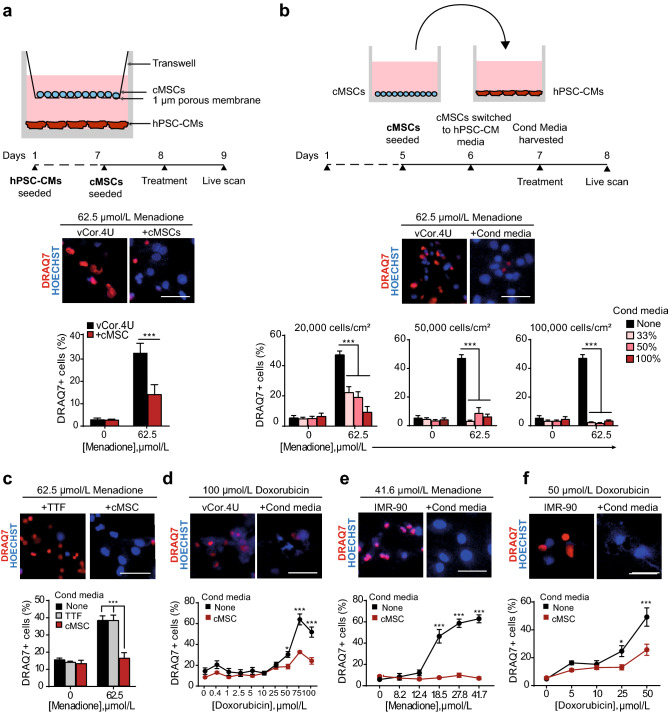
Mouse cMSC secretome suppresses cell death in human cardiomyocytes from pluripotent stem cells. (**a**) cMSC protect vCor.4U human ventricular myocytes from lethal oxidative stress in trans-well co-culture. Above, schematic representation and timeline. Middle, representative images of nuclear DRAQ7 staining after a menadione challenge. Below, bar graph of DRAQ7 uptake; n = 6. cMSC were prospectively sorted using Sca1 and the SP phenotype, and were 90% PDGFRα^+^. (**b**) cMSC-conditioned media protect vCor.4U human ventricular myocytes from lethal oxidative stress. Above, schematic representation and timeline. Middle, representative images as in (**a**). For the cultures illustrated, cMSC were seeded at 100,000 cells/cm^2^ and conditioned media used at a concentration of 50%. Below, bar graphs of DRAQ7 uptake, at the indicated cMSC seeding densities and media concentrations; n = 9. (**c**,**d**) Specificity and generality of protection in vCor.4U human ventricular myocytes. Above, representative images. Below, bar graphs of DRAQ7 uptake; n = 9. (**c**) Lack of protection from menadione by tail tip fibroblast-conditioned medium. (**d**) Protection from doxorubicin by cMSC-conditioned media. (**e**,**f**) cMSC-conditioned media was tested on IMR-90 hPSC-CMs, an independent human cardiomyocyte line. (**e**) Representative images and bar graph of DRAQ7 uptake after menadione. n = 12. (**f**) Representative images and bar graph of DRAQ7 uptake after doxorubicin; n = 9. For all panels: scale bar, 50 μm; data are shown as the mean ± SEM; *p < 0.05; ***p < 0.0001.

To identify mechanisms underlying the reduction of infarct size and prevention of heart failure conferred by intramyocardial injection of cMSC previously described, we used cells that were prospectively isolated using the same markers, Sca1^+^ SP cells^5^. Specifically, a pro-survival paracrine effect on cardiomyocytes was postulated. For a direct assessment of cMSC’s paracrine effects in a relevant human context, a co-culture system was established using human ventricular cardiomyocytes derived from pluripotent stem cells (vCor.4U, Ncardia)^20,21^, plated at the bottom of a two-chamber system, with Sca1^+^ SP stromal cells above (Fig. 1a, top). By this means, the “target” and “secreter” cell populations were separated by a microporous membrane that prevents cell migration but allows the transit of secreted factors. Co-cultures were exposed to menadione, a naphthoquinone that induces endogenous reactive oxygen species (ROS)^32^, instrumental mediators of ischemia–reperfusion injury as in myocardial infarction (Supplementary Fig. S1)^23,33,34^. As shown by the 56.5% inhibition of DRAQ7 uptake, co-culture with cMSCs protected the human cardiomyocytes from cell death after oxidative stress (p = 0.0007; Fig. 1a, bottom).

Previously, we showed that SP cells are enriched for PDGFRα which is synergistic with the SP status as a determinant of clonal growth and together define the highly clonogenic cardiac stromal cells with cardiovascular differentiation capacity after grafting^5^. As expected, PDGFRα was enriched in the expanded Sca1^+^ SP cells used for the present experiments (Supplementary Fig. S2A). Importantly, to exclude fortuitous effects from the genotoxicity of Hoechst 33342 and ultraviolet irradiation required for preparative sorting of SP cells, we also tested short-term pools of PDGFRα^+^ CD31^−^ Sca1^+^ adult mouse cardiac cells, expanded for 5–10 passages. cMSCs produced by this alternative protocol were highly enriched for the SP phenotype (Supplementary Fig. S2B,C) and provided comparable protection in co-culture to hPSC-CMs (Supplementary Fig. S2D).

To explore cMSC’s paracrine signalling further, the cells were plated at increasing densities, and the respective media were added to vCor.4U cardiomyocytes at three concentrations at the time of menadione treatment (Fig. 1b, top). As shown by the dose-dependent reduction of DRAQ7^+^ uptake, cMSC-conditioned media suppressed human cardiac muscle cell death, in proportion to donor cMSC number and the conditioned media concentration, reaching > 85% inhibition of cardiomyocyte death at ~ 50,000 cMSC/cm^2^ (p < 0.0001; Fig. 1b, bottom). In a direct side-by-side comparison, the conditioned media from tail tip fibroblasts conferred no statistically significant protection (Fig. 1c). In contrast to this potent reduction of cardiomyocyte death, the cMSC-secretome did not affect DNA synthesis in the vCor.4U cells, as measured by EdU staining after 48 h of treatment with the conditioned media (Supplementary Fig. S2E).

To test whether cMSC-conditioned media might disrupt human cardiomyocyte death more generally, vCor.4U hPSC-CMs were treated with the cardiotoxic anthracycline, doxorubicin^22^. As with menadione, cardiomyocyte death triggered by doxorubicin was markedly suppressed by cMSC-conditioned media (p < 0.0001; Fig. 1d).

Next, IMR90-derived cardiomyocytes^35^ were likewise tested as a target for cardiac protection, as an independent, complementary hPSC-CM line, derived from a different human donor, tissue source, method of generation, method of differentiation, and maintenance medium. Immunostaining against the cardiac-specific marker α-actinin, demonstrated a purity of 96.2 ± 2.6% in IMR90-CMs (Supplementary Fig. S3A). RNA-seq analysis of the IMR90-CM transcriptome indicates a level of maturity at least comparable to hPSC-CMs previously used for translational studies^22,35^ (Supplementary Fig. S4; Table S5): (1) no residual expression of pluripotency and cardiac mesoderm genes (*NANOG*, *SOX2*, *POU5F1*, *EOMES*, *TBXT*, *KDR*, *PDGFRA*); (2) highly robust expression of sarcomeric genes (*ACTC1*, *MYH7*, *TNNC1*,*TNNT2*)*;* (3) presence of transcripts encoding for SERCA2 *(ATP2A2);* (4) expression of major adult L-type calcium channels (e.g. *CACNA1C*) as well as the major sodium (*SCN5A*) and potassium (*KCNH2*) cardiac channels; (5) absence or low expression of fibroblast markers (*DCN*, *DDR2*) and (6) expression of typical cardiac transcription factors such as *NKX2-5* (Supplementary Fig. S3B–D)*.* Furthermore, hPSC-CMs under these conditions of culture, have progressed to a mature, mitogen-resistant state, consistent with the reported post-mitotic phenotype in other studies, even in 2D culture^36,37^. Consistent with this empirical finding, RNA-seq analysis shows: (1) high expression of the tumor suppressor gene *RB1* versus its relative *RBL1 (p107)*, which underlies the developmental switch to a post-mitotic phenotype^38–40^; (2) high expression of *CDKN1A* encoding for Cdk inhibitors p21 and the transcript for p27, *CDKN1B* (Supplementary Fig. S3E). Exploratory studies suggested a possible trophic effect of conditioned medium on hPSC-CMs as shown by the increase in *NPPA* expression, however, this increase was only two-fold, much smaller than in bona fide hypertrophy; also *NPPB* expression increased just 1.5 fold. These changes were not accompanied by down-regulation of *MYH6* nor induction of *ACTA1* and *MYL4* (Supplementary Fig. S3F)^41–44^.

Consistent with the results in vCor.4U cardiomyocytes, cMSC-conditioned media protected IMR90-derived CMs from cell death triggered by either menadione or doxorubicin, as well as by imatinib, a cardiotoxic anti-cancer tyrosine kinase inhibitor (Fig. 1e,f; Supplementary Fig. S6A). Partial protection was seen, even when giving cMSC-conditioned medium one hour after oxidative stress (Supplementary Fig. S6B). Thus, mouse cMSCs confer a potent pro-survival benefit in human cardiomyocytes derived independently from two pluripotent cell lines, and three independent death signals. Taken together, these data support the conclusion that cMSC-dependent cytoprotection is generalisable to clinically relevant compounds, beyond just the use of menadione as a model for oxidative stress.

### cMSC-conditioned medium down-regulates apoptosis-related genes and preserves cardiac structural genes in lethally stressed human cardiomyocytes

**Figure 2:**
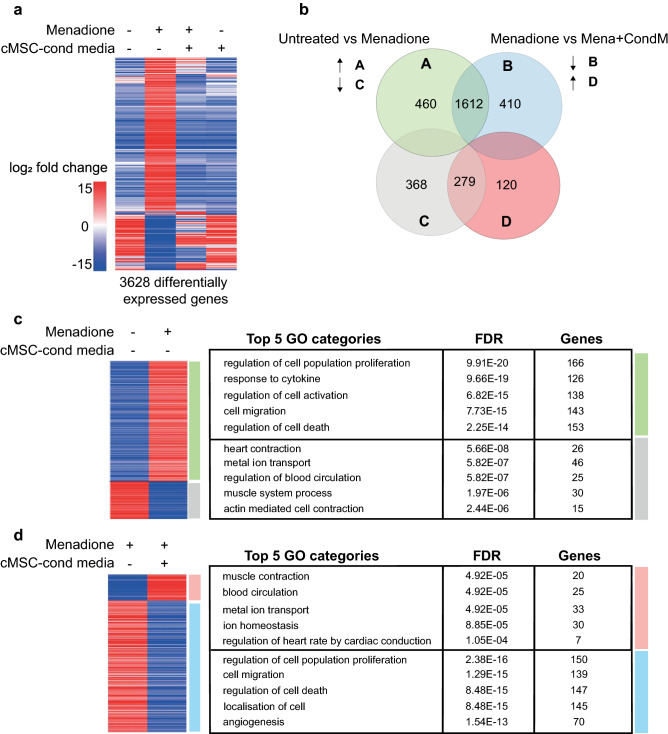
RNA-Seq analysis of IMR90-cardiomyocytes ± menadione and cMSC-conditioned medium. (**a**) Heatmap of the 3,628 differentially expressed genes (log_2_fold change > 2, p value < 0.05) across the four IMR90-CM treatment groups (n = 3), shown by unsupervised cluster analysis. Oxidative stress was induced for 24 h with 20 μM menadione with and without cMSC-conditioned medium treatment. The heatmap was created in SeqMonk using Pearson’s Correlation clustering for the genes (y-axis). (**b**) Venn diagram showing overlapping genes shared in the pairwise comparisons indicated. Green, up-regulated genes vs untreated; grey, down-regulated genes vs untreated; blue, up-regulated genes vs menadione-stressed; red, down-regulated genes vs menadione-stressed. (**c**,**d**) Curated heatmaps and tables of GOs for the changes induced by (**c**) menadione or (**d**) cMSC-conditioned medium. Tables include top 5 non-redundant GOs, from the ToppGene “Biological Process” database. For a full list of the generated GOs see Supplementary Fig. S7. These data were deposited on SRA public repository with accession number PRJNA629893.

To explore the protection of hPSC-CMs from menadione mediated by cMSC-conditioned medium, unbiased RNA-sequencing was performed on the following groups of IMR90-derived CMs: (1) untreated, (2) treated for 24 h with menadione, (3) treated with cMSC-conditioned medium and (4) treated together with menadione and cMSC-conditioned medium (Fig. 2a). A total of 3,628 genes were differentially expressed across the four groups (log_2_fold change > 2 and p value < 0.05), with the majority of these changes occurring in the transcriptome of menadione-treated myocytes relative to their basal expression (2,719 differentially expressed genes; Fig. 2a). Gene Ontology (GO) analysis of the transcripts induced in stressed myocytes identified categories directly relevant to the observed protection including “*regulation of cell death”* (153 genes, FDR 7.56E−19), along with other GO categories relevant to paracrine circuits like “*response to cytokine*” (126 genes, FDR 9.66E−19), “*cell activation*” (138 genes, FDR 3.10E−17) and “*regulation of cell population proliferation*” (166 genes, FDR 2.25E−14; see Fig. 2c; Supplementary Table S7). Changes related to cell death included the induction of genes encoding proteins with proven adverse functions in cardiac muscle cell survival, such as death domain receptors (*TNFRSF8*, *TNFRSF10D*, *TNFRSF18*)^45^ and DNA damage-inducible proteins and growth arrest mediators (*GADD45B*, *GADD45G*)^46,47^. Notable was the upregulation of *PMAIP1/NOXA*, whose product regulates mitochondrial membrane permeabilization and the release of apoptogens^48^. Given that cardiomyocyte function is severely affected under conditions of oxidative stress, it was not surprising to detect downregulation of genes related to GOs of “*heart contraction”*’ (FDR 5.66E−08) and “*metal ion transport*” (FDR 5.82E−07), encompassing transcripts encoding major cardiac ion channels (*CACNA1G*, *KCNJ4*, *KCNJ5*, *SCN1A*, *SCN5A*), transporters (*ATP1A2*), and sarcomeric genes (*MYH7*, *TNNI2*) (Fig. 2c; Supplementary Table S7)^46,47^.

Treatment with cMSC-conditioned media broadly interfered with menadione-dependent gene expression in the lethally-stressed cardiomyocytes, with 2,421 transcripts affected (Fig. 2a). Conditioned medium prevents the expression of ~ 80% of the menadione-induced genes and it rescues > 40% of the transcripts that were downregulated in the stressed cardiomyocytes (Fig. 2b). Specifically, the cMSC secretome prevented the expression of most genes associated with the GO cluster “*regulation of cell death*” (FDR 8.48E−15), while preserving expression of genes in GOs related to cardiac function such as “*muscle contraction*” (FDR 4.92E−05) (Fig. 2d; Supplementary Table S7). Intriguingly, treating menadione-stressed myocytes with the cMSC secretome inhibited the expression of transcripts for oxidative stress response factors (*ETS1*, *MMP3*, *FOS, CYP1B1, ANXA1*)^46,47,49^, pro-death signals (*TNFRSF8*, *TNFRSF10D*, *TNFRSF12A*)^45,47^ and Toll-like receptors (*TLR4, TLR6*), which are part of the innate immunity circuit in cardiac muscle mediating some forms of cardiomyocyte death. Also, notable was interference with several transcripts encoding pro-apoptotic proteins known to affect cardiomyocytes (*ALK7, BCL10, GADD45B, PHLDA*)^46,47^ and others, not yet studied in the context of cardiac cell death, that are recognized mediators of apoptosis in other cell types (*FOXL2, INHBA, CASP4, IRF1, PMAIP1, PLEKHN1*)^50–53^. In summary, transcriptomic analysis and, specifically, the down-regulation of death- and apoptosis-related genes by cMSC-conditioned medium suggests that the potent rescue of human cardiomyocyte survival depends on attenuation of one or more transcriptional circuits for apoptosis.

### The mouse cMSC secretome blocks apoptosis and the dissipation of mitochondrial potential induced by menadione

**Figure 3:**
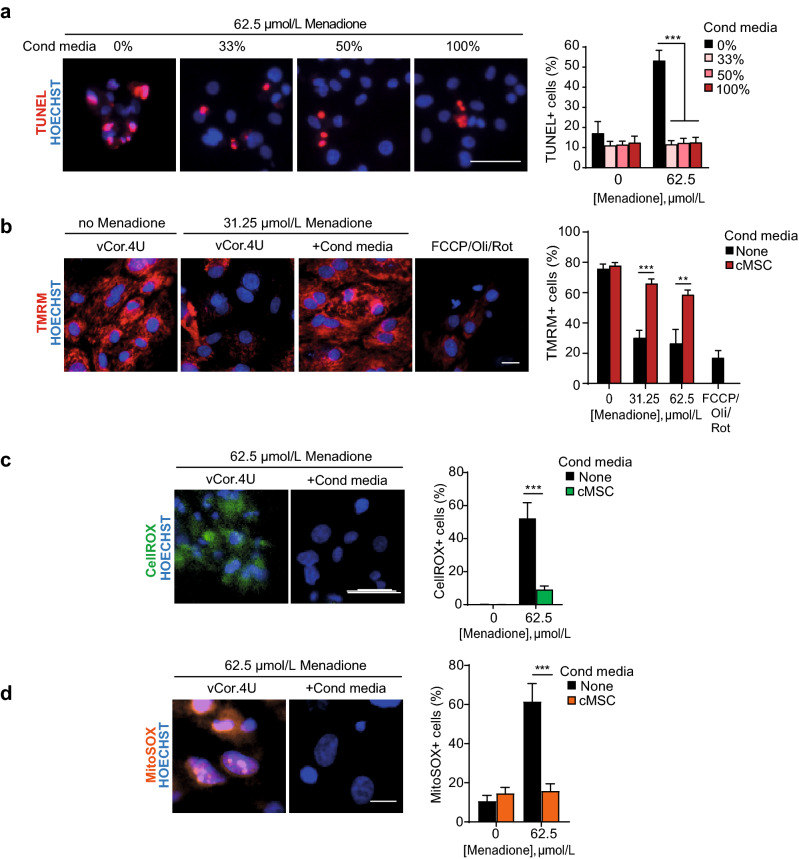
Mouse cMSCs’ secretome blocks human ventricular myocyte apoptosis and the dissipation of mitochondrial potential induced by menadione. (**a**) Representative images and bar graph of TUNEL^+^ vCor.4U human ventricular myocytes, 24 h after menadione ± cMSC-conditioned media at the concentrations shown. n = 9. (**b**) Representative images and bar graph of mitochondrial TMRM in vCor.4U hPSC-CMs, stressed with menadione ± cMSC-conditioned media. Carbonyl cyanide 4-(trifluoromethoxy) phenylhydrazone (FCCP), oligomycin (Oli) and rotenone (Rot), uncouplers of oxidative phosphorylation, were used as controls. n = 7. (**c**) Representative images and bar graph of CellROX in vCor.4U hPSC-CMs 8 h after menadione ± cMSC-conditioned media. n = 12. (**d**) Representative images and bar graph of MitoSOX 4 h after menadione ± cMSC-conditioned media. n = 7. For all panels: scale bar 50 μm; data are shown as the mean ± SEM; *p < 0.05; ***p < 0.0001.

Given the transcriptomic prediction above, cardiac DNA fragmentation was evaluated by the TUNEL assay to test empirically both the prevalence of apoptosis following menadione treatment and whether this is blocked by cMSCs. Human cardiomyocytes treated with menadione ± conditioned media at three different concentrations were compared to untreated cells and tested by TUNEL assay after 24 h. cMSC-conditioned media reduced DNA fragmentation to the basal levels seen in unstressed cells, even with 33% conditioned media, the lowest concentration tested (p < 0.0001; Fig. 3a). As the classically defined “intrinsic” apoptotic pathway hinges on mitochondrial depolarisation, we monitored mitochondrial membrane potential using the cell-permeant dye, tetramethylehodamine methyl ester perchlorate (TMRM; Fig. 3b; Supplementary Fig. S6C). In the absence of oxidative stress, vCor.4U and IMR-90 cardiomyocytes showed abundant active mitochondria, as expected. Following oxidative stress, loss of the TMRM signal was confirmed. Conversely, cMSC-conditioned media preserved mitochondrial membrane potential in each of these two independent human cardiomyocyte lines (p = 0.0004 and p < 0.0001, respectively, in vCor.4U- and IMR-90-derived cardiomyocytes). Given known actions of menadione in both the cytosol and mitochondrial compartments^54^, and the role of mitochondria in feedback loops that amplify ROS production, we next tested whether cMSC-conditioned media can attenuate the formation of ROS induced by menadione. CellROX staining demonstrated that cMSC-conditioned media strongly reduces whole-cell ROS production in hPSC-CMs (Fig. 3c; Supplementary Fig. Fig. S6D,E), resembling the impact of bone marrow- or iPSC-derived MSC signalling to rodent cardiomyocytes^55^. Using MitoSOX, which detects mitochondrial superoxides specifically, we further showed that cMSC-conditioned media blocks superoxide production within the mitochondrial compartment (Fig. 3d). These data suggest that cMSC-conditioned media impinge on the mitochondria-dependent apoptotic pathway in human cardiomyocytes, at least in part through interference with sustained ROS accumulation.

### An exosome-independent mechanism mediates the observed protection by mouse cMSCs

**Figure 4:**
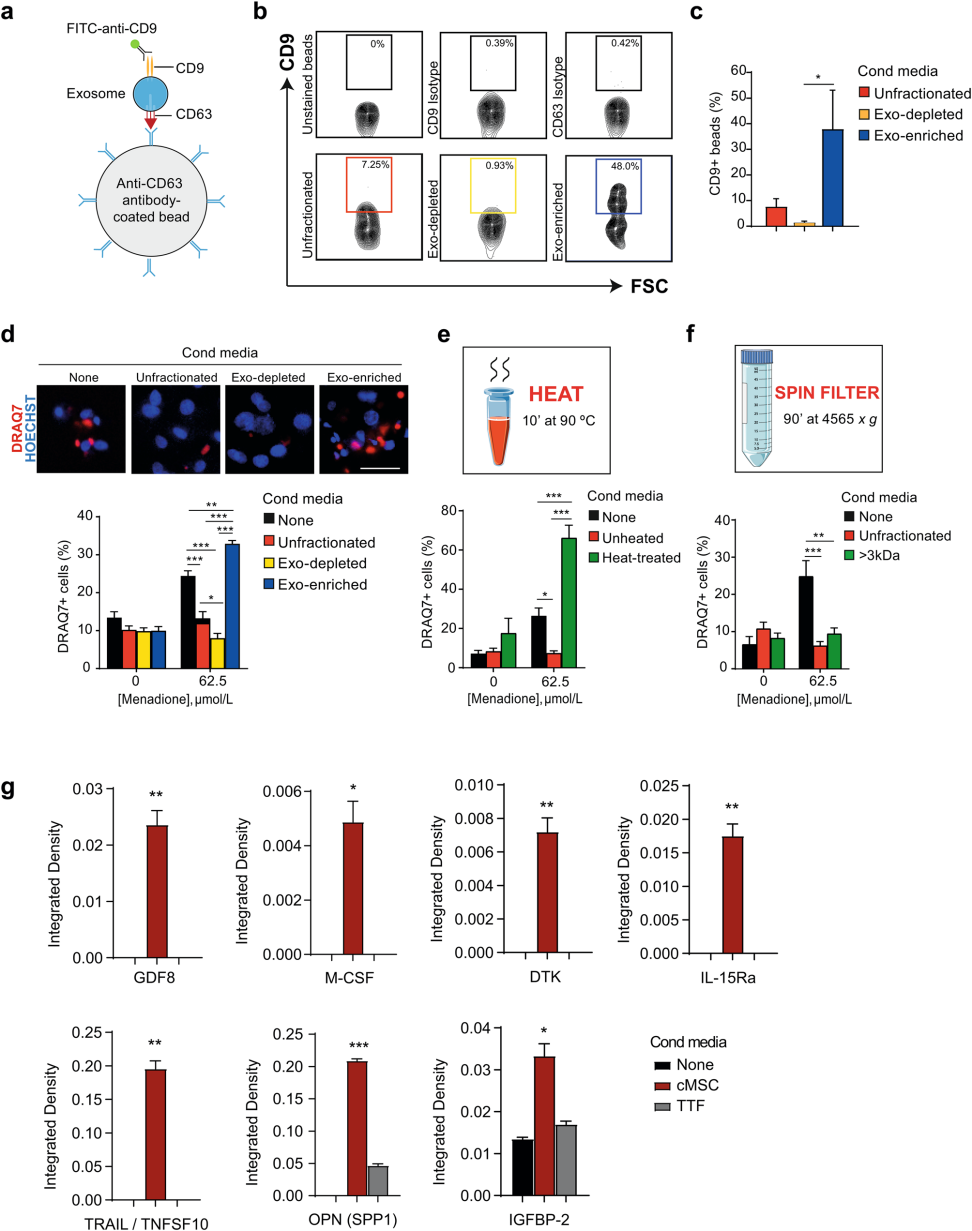
An exosome-independent mechanism mediates the observed protection of human cardiomyocytes by mouse cMSC. (**a**) Schematic of exosome capture and detection using anti-CD63-conjugated latex beads plus FITC-anti-CD9. (**b**,**c**) Bead-exosome complexes were analysed by flow cytometry. (**b**) Representative contour plots are shown. The gate defines the CD63^+^CD9^+^ exosomes. The top row shows controls for staining and the bottom row the proportion of exosomes in unfractionated conditioned media versus the depleted (exo-depleted) and enriched (exo-enriched) fractions. *FSC* forward scatter. More than 5,000 beads were scored for each condition shown. (**c**) Bar graph of CD9^+^ depletion and enrichment; n = 3. (**d**) DRAQ7 uptake in vCor.4U human ventricular myocytes after menadione ± cMSC-conditioned media or the indicated fractions. Above, representative images. Scale bar, 50 μm. Below, bar graph of DRAQ7 uptake; n = 10. (**e**) Thermostability testing of cMSC-conditioned media. Bar graph of DRAQ7 uptake; n = 9. (**f**) Size-fractionation of cMSC-conditioned media. Bar graph of DRAQ7 uptake; n = 6. For all panels, data are shown as the mean ± SEM. *p < 0.05; **p < 0.001; ***p < 0.0001. (**g**) Bar graphs of cytokine levels, using low-density membrane arrays, for the factors enriched in cMSC-conditioned medium vs medium only or TTF-conditioned medium. Results are image analysis of integrated density, normalised to the average of anti-streptavidin and anti-HRP controls for each membrane. Enlarged versions of the arrays can be found in Supplementary Fig. S9. Data are mean ± SEM; n = 2; *p < 0.05; **p < 0.01; ***p < 0.001. Unpaired one-tailed t test between cMSC and medium or cMSC and TTF. Graphics were created using Servier Medical Art website, a free medical image database with a licence under Creative Commons Attribution 3.0 Unported License (https://creativecommons.org/licenses/by/3.0/).

In several noteworthy studies, the paracrine benefits of cell therapy products like certain reported mouse and human cMSCs, cardiosphere-derived cells, MSCs, and PSC-CMs have been attributed to cell–cell communication by exosomes^56^. To assess whether exosomes from the well-characterised cMSCs used here mediate the observed protection of cardiomyocytes, hPSC-CMs challenged with menadione were treated with exosome-depleted or -enriched cMSC-conditioned medium, versus unfractionated cMSC-conditioned medium as the control. Exosomes were isolated by ultracentrifugation and their resulting depletion or enrichment was validated by flow cytometry^57^: isolated exosomes were captured using CD63-coated latex beads and detected with a fluorescently conjugated antibody against CD9 (Fig. 4a,b). As previously reported for this procedure^57^, exosomes were enriched more than 20-fold, comparing the respective fractions (Fig. 4b,c). Whereas exosome-depleted medium conferred protection at least equal to that of the unfractionated medium control, myocyte survival was not improved by exosome-enriched medium compared to the unfractionated media and slightly worsened compared to the standard medium (Fig. 4d). Similar results were obtained by complementary procedures, using an independent spin-column-based method for exosome precipitation and size-fractionation (Supplementary Fig. S8). Thus, under the specific conditions tested here, mechanisms alternative to exosomes likely mediate the observed protection of human cardiomyocytes. Consequently, the possibilities include growth factors, cytokines, chemokines, non-vesicular RNAs, lipids and low-molecular weight metabolites. Therefore, to obtain inferences on the nature of the factors mediating the protective effects, thermal stability testing was performed. Paracrine protection depends on thermolabile molecules and on molecules exceeding 3 kDa in size, for both hPSC-CM lines (Fig. 4e,f; Supplementary Fig. S6F,G). The increase in cell-death in heat-treated conditioned-medium is likely due to the depletion of essential nutrients. Together, these results provide evidence against a metabolite as the responsible mediator and are consistent, instead, with protection by one or more protein factors.

An exploratory screening of the cardioprotective cMSC secretome was performed using cytokine membrane arrays pre-printed with capture antibodies against 308 mouse proteins. Conditioned medium from cMSC was compared to non-protective TTF and IMR90 basal medium. Statistically significant enrichment was seen for just 7 proteins (DTK, GDF-8, IGFBP-2, IL-15Rα, M-CSF, OPN and TRAIL) in cMSC-conditioned medium compared to the two non-protective controls (Fig. 4g; Supplementary Fig. S9). Of these, M-CSF, IL-15Ra and IGFBP2 have each been implicated in pro-survival signalling in the heart and specifically have been shown to inhibit cardiomyocyte apoptosis^58–60^. GDF-8 best known as an inhibitor of skeletal muscle growth, regulates energy homeostasis in the mouse heart, prevents cardiac hypertrophy and inhibits cardiomyocyte proliferation in zebrafish, with no demonstrated impact on apoptosis^61,62^. The DTK receptor tyrosine kinase was recently shown to undergo a cleavage step and have an anti-apoptotic effect in cancer cells, though the role of the released extracellular domain is still unclear^63^. Two pro-apoptotic mediators that commonly promote cell death but have highly variable consequences in cardiomyocytes, including suppressive effects on cell death *(OPN*, *TRAIL*)^45,64–66^ were also enriched in the cMSC secretome. Together, our results suggest that a combination of factors is likely mediating paracrine signals whose net effect is cardio-protection.

### Human cardiac stromal cells protect human cardiomyocytes

**Figure 5:**
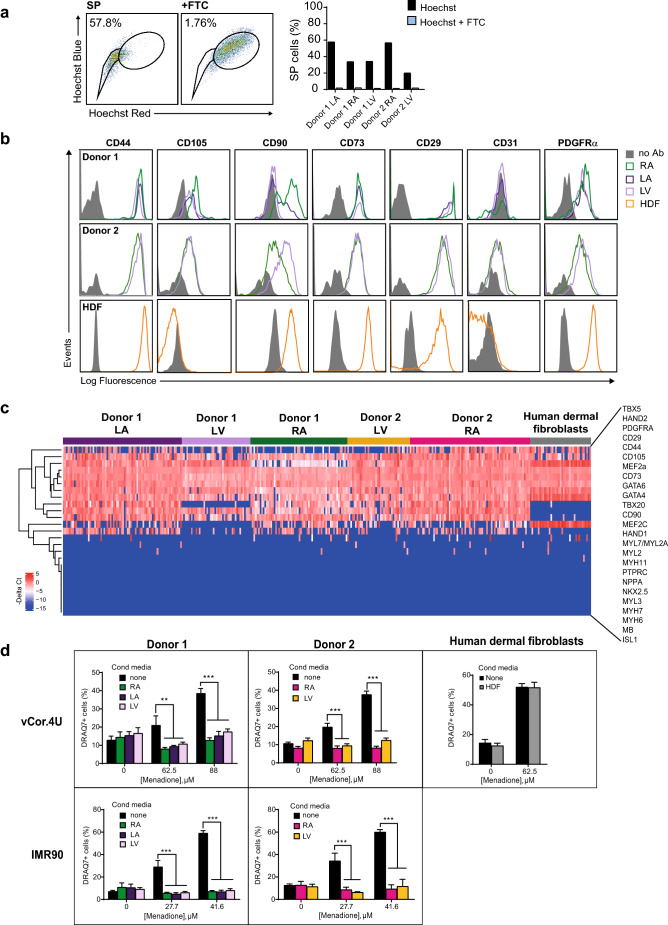
Human cardiac stromal cells protect human cardiomyocytes. (**a**) Human cardiac stromal cells are enriched for the SP phenotype. Left, representative dot plots of SP staining for the left atrium of Donor 1. FTC, ABCG2 inhibitor Fumitremorgin C. Right, bar graphs showing consistent enrichment for SP cells in five human cardiac stromal cell populations from two donors. (**b**) Flow cytometry comparing surface marker expression in the human cardiac stromal cell populations and human dermal fibroblasts (HDFs). Note the absence of CD105 in HDFs. (**c**) Single-cell qRT–PCR comparing the co-expression of selected genes in human cardiac stromal cells and HDFs. The heatmap shows expression as − ΔCt values (blue, low; red, high). Genes are ordered based on hierarchical clustering. n = 39–72 cells for each sample illustrated. See also Fig. S3 in the supplementary data. (**d**) Human cardiac stromal cells from the donors and chambers shown all suppress cell death induced by menadione in vCor.4U (top) and IMR-90 (bottom) human cardiomyocytes; HDFs (right) had no effect. Bar graph, mean ± SEM; vCor,4U: n = 6; IMR-90: n = 9; **p < 0.001; ***p < 0.0001. *RA* right atrium, *LA* left atrium, *LV* left ventricle.

PDGFRα^+^ cells exist in the adult human cardiac interstitium^67^, though their functional properties are largely unexplored. To test for paracrine protection of human cardiomyocytes in a wholly human model, we isolated and expanded human cardiac stromal cells. Enrichment for SP cells was confirmed by flow cytometry in the absence and presence of fumitremorgin C, a potent inhibitor of the ABCG2 transporter, the pump responsible for the extrusion of Hoechst 33342 (Fig. 5a)^68^. As predicted, expression of PDGFRα and MSC markers (CD29, CD44, CD73, CD105) was detected by flow cytometry (Fig. 5b) and by single-cell qPCR (Fig. 5c). Cardiac transcription factors were co-expressed uniformly (*GATA4, GATA6, HAND2, MEF2A, MEF2C, TBX20*), but no later differentiation markers were observed (Fig. 5c; Supplementary Fig. S10). Typically, human dermal fibroblasts, used as controls, were enriched for *CD90* and did not express *GATA4*, *GATA6* or *TBX20* (Fig. 5c; Supplementary Fig. S10). Hence, by these combined criteria, these human cells strongly resemble the mouse cells conferring cardiomyocyte protection in Figs. 1, 2, 3 and 4^5^. In addition, they resemble certain human cardiac mesenchymal cells reported previously^11^, albeit without need of histone deacetylase depletion, and correspond better, by this signature, to the cardiac-resident MSC^27^ and closely related populations^6,13^. Samples from all five patients tested suppressed cell death induced by menadione in the two human cardiomyocyte lines (p < 0.0001; Fig. 5d). Human dermal fibroblasts, used as an irrelevant control, had no effect. In summary, we isolated and expanded a population of human cardiac stromal cells with surface properties, molecular signatures, side population phenotype, and protective potency substantially comparable to those of the cardioprotective mouse cMSCs. To our knowledge, this is the first evidence that paracrine signals from human cardiac stromal cells can suppress human cardiac muscle cell death.

### Conditioned media from mouse cMSC is sufficient to suppress local cardiomyocyte apoptosis after myocardial infarction in mice

**Figure 6:**
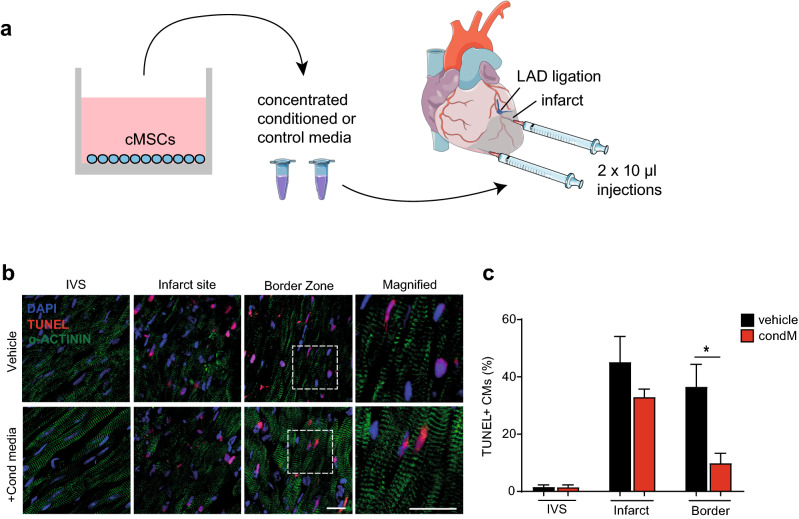
Conditioned media from mouse cMSC suppress cardiomyocyte apoptosis after mouse myocardial infarction. (**a**) Schematic representation of cMSC-conditioned medium production, concentration and intramyocardial injection after LAD ligation. Concentrated conditioned or control media were injected into the infarct border zone, one site ~ 1 mm beneath the suture and a second site more apical. Images modified from Servier Medical Art website, a free medical image database with a licence under Creative Commons Attribution 3.0 Unported License (https://creativecommons.org/licenses/by/3.0/). (**b**) Representative immunohistochemistry images of TUNEL staining, 24 h after myocardial infarction. An α-actinin antibody was used to demarcate cardiomyocyte identity. Scale bars 20 μm. See also Supplementary Fig. S11. (**c**) Bar graph of TUNEL staining in cardiomyocytes in the remote myocardium (intraventricular septum, IVS), infarct site, and border zone. Data are shown as the mean ± SEM. n = 3; *p < 0.05.

In order to test whether cMSC-conditioned medium, as a cell-free product, has a protective effect on cardiomyocytes in vivo, we performed permanent ligation of the left coronary artery inducing myocardial infarction in C57Bl/6 mice^5^. Conditioned media produced by cMSC and unconditioned control media were concentrated and injected into the border zone at the time of injury (Fig. 6). To confirm intramyocardial delivery and spatial distribution, the conditioned and control media were co-injected with a lipophilic fluorescent carbocyanine dye (Supplementary Fig. S11). Hearts were collected 24 h post-MI, when cell death is virtually complete^69^, and cardiac DNA fragmentation detected by terminal deoxynucleotidyl transferase dUTP nick end-labeling (TUNEL) was used as a selective measurement of cell death through apoptosis (Fig. 6). As expected, a significant reduction (> 70%) of cardiomyocyte apoptosis was detected in the injected border zone (p = 0.02), with no significant reduction in the core region of infarction, distant from the injection site. In summary, conditioned media from cMSC is sufficient, not only, to promote human cardiomyocyte survival, but also, as proof of principle in vivo, to suppress local cardiomyocyte death in mouse myocardial infarction.

## Discussion

Organ-level benefits that have been ascribed, provisionally, to paracrine signals from grafted cells include scar regression, myocyte proliferation, resident stem cell activation, immunomodulation, and cardiomyocyte protection, among others^5,7–10,16^. As with cardiac target validation for other pathobiological and therapeutic circuits, these premises will likely benefit from rigorous testing in a human context, not animal models alone^20,21,70^. The irregular availability of human cardiomyocytes from biopsies or explanted hearts and the inability of cardiomyocytes from these sources to expand in culture have made the human cardiac phenotype inaccessible for routine and scalable experimental therapeutics. The advent of hPSC-CMs has overcome this long-standing impasse, albeit applied thus far more to safety pharmacology^21^, drug-induced cardiomyopathies^22^, and hereditary disorders^71^ than to the globally most prevalent forms of acquired heart disease including ischemic heart disease. Targeting cardiac muscle cell number to reduce infarct size—further preventing cell death as an adjunct to reperfusion—is an urgent therapeutic need to improve survival post-MI^16^, as infarct size predicts the risk of heart failure and 1 year all-cause mortality^72^. Thus, considering the past failures of promising interventions for cardiomyocyte protection, all based on animal models alone, we emphasize the use of a human model, instead, to establish preclinical efficacy and explore the mechanisms of benefit.

Here, we demonstrate the utility of hPSC-CMs as a human platform to investigate paracrine signals that underpin cardioprotection by cardiac cell therapy^16^. Cell death induced by oxidative stress, apoptotic DNA fragmentation, the dissipation of mitochondrial membrane potential, and mitochondrial superoxide formation were all suppressed by cMSC-conditioned medium, using murine cMSC that prevent adverse remodelling and heart failure in infarcted mice. As further confirmation of the cMSC-dependent paracrine protective effect, conditioned medium was shown to reduce cardiomyocyte apoptosis in mouse myocardial infarction, within the injected region. Intramyocardial delivery of conditioned media results in a localised diffusion as shown by the tracking agent that was co-injected. Therefore, to test improvement of cardiac function, tissue engineering approaches will be required to ensure broader and controlled delivery of conditioned media in space and time.

Notably, cMSC-conditioned media also protected human cardiomyocytes from death induced by two clinically relevant cardiotoxic drugs (doxorubicin and imatinib; Fig. 1d,f; Supplementary Fig. S6A), suggesting that the cytoprotecive effect might involve multiple pro-survival mechanisms or, perhaps more likely, a shared mediator such as whole-cell ROS. Consistent with the hypothesis that protein factors mediate the paracrine protection, conditioned media were concentrated using columns with a cutoff of 3 kDa for in vivo experiments, following substantiation of this fraction’s effect in the human cardiomyocytes (Fig. 4f).

Although hPSC-CMs represent a scalable human platform for cardiac drug discovery reproducing many relevant features of cardiomyocytes’ function, a few cautionary notes need to be highlighted. Cells used here include vCor4U that enrich for ventricular cardiomyocytes^23^, the relevant cells in a model of ischemic injury. Our RNA-seq data indicate that IMR-90 express cardiomyocytes’ structural and functional genes in the absence of reprogramming genes, a proliferation signature or fibroblast markers. Notably, recent work demonstrates the predictive power of hPSC-CMs even in routine 2D culture^21,23,24,73^. Nevertheless, improvement of structural and functional maturity, as well as chamber specificity in vitro remains a highly sought goal. Currently improvement in maturation and specificity is achieved by the use of 3D human engineered heart tissue, mechanical or electrical conditioning, and heart-on-chip technologies although throughput and/or accessibility of these methods remain limiting factors^74–78^. Interestingly, low sensitivity to hypoxia/reoxygenation was previously reported for hPSC-CMs^23,79^, and in our experimental setting, although differences were observed between the lines studied in their sensitivity to menadione, the paracrine protective effect was consistent and comparable. Interestingly, higher susceptibility of hPSC-CM to ischemic damage was linked to a polymorphism of ALDH2 and, unsurprisingly, screening a single commercial line failed to capture the known cardiotoxicity of rosiglitazone^80,81^. Thus, it is plausible that patient-specific susceptibility might contribute to the differences in response to oxidative stress together with variability in the levels of maturity and/or chamber specificity. Future translational drug and target validation will need to include more ample demographic features such as gender and ethnic background. Studying cMSCs’ paracrine effects in vitro provides a clean model to dissect cardiomyocyte-specific protective mechanisms, however, as in any in vitro system the effects on the cellular landscape and intercellular networks in the tissue will not be captured by this model. Notably, here, we provided evidence that the protective effect of cMSCs-conditioned media on cardiomyocytes is reproduced in the myocardium.

Of immediate benefit here, we validate a workable model to investigate extracellular signals that augment human cardiac muscle survival, demonstrating that the secretomes of mouse and human cMSC drastically suppress human cardiomyocyte death. This provides an accessible, scalable, translationally relevant platform toward future therapeutic cell-free strategies, based on the use of conditioned media but also towards more refined interventions via the identification of specific extracellular factors or their downstream mediators. The magnitude of protection conferred to human pluripotent cell-derived cardiomyocytes under these reductionist conditions, while just a first step, provides the most direct support yet for the seminal hypothesis that heart-derived cells protect human cardiomyocytes from lethal stress in clinical settings^16^.

Moreover, the assays detailed here are well-suited to high-throughput, high-content imaging with limited numbers of human myocytes per well and consequently to the large scale needed to deconvolute conditioned media in future gain- and loss-of-function studies. More comprehensive sample collection and deep, unbiased profiling should help inform the quest for a consensus secretome that tracks with human cardiac muscle cell protection and whether this changes in cardiac disease.

Under our experimental settings, we see an exosome-independent mechanism of protection. The implication of exosomes in the cardio-protective effects of our secretor cMSCs has not been previously tested and no study to our knowledge has shown exosome-mediated protection of human cardiomyocytes from apoptotic death. This might also explain the slight increase observed in hPSC-CM death in the presence of the exosome-enriched fraction.

In addition, the data are consistent with the possibility that conditioned media, if suitably optimized, could be used as a cell-free product to suppress cardiomyocyte death induced by ischemic injury, as a potential adjunct to merely restoring blood flow. The potential use of conditioned media as cell-free therapy has several noteworthy advantages over cells in terms of translatability, including most obviously, the lack of histocompatibility issues. A translational implementation could likely require a biomaterials approach, to achieve more effective delivery and widespread effect. Advances in the development of sophisticated scaffolds make the controlled release of conditioned media or recombinant factors a tangible objective even for the heart^82,83^.

## Methods

Animal procedures were performed with UK Home Office approval (PL 70/6806, 70/7880) and conform to the UK Animals (Scientific Procedures) Act, 1986, incorporating Directive 2010/63/EU of the European Parliament.

### cMSC isolation

Hearts from C57Bl/6 adult male mice (8–13 weeks old; Charles River) were dissociated using 100 μg/ml Liberase and 50 μg/ml DNAse I (Roche)^5^. The cardiomyocyte-depleted preparation was filtered through a 70 μm mesh (BD Falcon). Hematopoietic lineage (Lin) depletion was performed using an AutoMACS Pro Separator (Miltenyi Biotec). To resolve SP and non-SP cells, staining with 5 μg/ml Hoechst 33342 (Sigma-Aldrich) was performed^5^. Adult mouse cardiac Lin^-^/Sca1^+^/SP cells (Supplementary Fig. S1) and Lin^-^/Sca1^+^/PDGFRα^+^/CD31^-^ cells (Supplementary Fig. S1) were selected by FACS^5^.

### Cell culture

cMSC were expanded in cMSC-mantainance medium [35% Iscove’s modified Dulbecco’s medium (IMDM, Invitrogen), 32.5% Dulbecco’s modified Eagle’s medium (DMEM, Invitrogen), 32.5% Ham’s F12 (Invitrogen), 1.3% B27 supplement (Invitrogen), 2 mmol/l l-glutamine (Invitrogen), 1 × Antibiotic–Antimycotic (Invitrogen), 0.14 mmol/l 2-mercaptoethanol (Sigma-Aldrich), 3.5% bovine growth serum (Hyclone), 6.5 ng/ml recombinant human epidermal growth factor (Peprotech), 13 ng/ml recombinant human fibroblast growth factor-basic (Peprotech), 0.0005 U/ml thrombin (Roche) and 0.65 ng/ml cardiotrophin-1 (Cell Sciences)]^5,84^. Cells were cultured on 50 μg/ml collagen type I (BD Bioscience) -coated plates, detached using Trypsin-0.25% EDTA (Gibco), and counted using a Vi-CELL XR (Beckman Coulter). cMSC were used at passage 16–30. Human cMSC were propagated by the identical method and used at passage 2–8.

Mouse tail tip fibroblasts (TTFs) were generated by mechanical and enzymatic dissociation. Tails of 30 adult C57BL/6 mice (Charles River) were minced into 1–3 mm pieces in pre-warmed 0.25% Trypsin–EDTA (Gibco) and incubated for 30 min at 37 °C. Tissue fragments were transferred into 0.1% gelatin (Sigma-Aldrich)-coated 10 cm dishes with 9 ml of DMEM containing 15% fetal bovine serum (Gibco) and 2 mmol/l l-glutamine, then were incubated for a week at 37 °C in 2% O_2_ to allow fibroblast outgrowth and attachment. At 30–50% confluency, the cells were detached using 0.25% Trypsin–EDTA (Gibco) and maintained until passage 8–10. Human dermal fibroblasts (Promocell) were maintained in Fibroblast Basal Medium 2 (PromoCell) plus Fibroblast Growth Medium 2 Supplemental Pack (PromoCell), containing fetal calf serum, recombinant human basic fibroblast growth factor and recombinant human insulin. Cells were passaged with 0.25% Trypsin–EDTA. Conditioned media from TTFs and human dermal fibroblasts were generated by the same protocol used for cMSC.

Human ventricular PSC-CMs (vCor.4U; Ncardia) were plated at 100,000 cells/cm^2^ in half-area 96-well microclear black-bottom plates (Greiner) coated with 50 μg/ml fibronectin and maintained in Cor.4U Complete Culture Medium (Ncardia). IMR-90-derived cardiomyocytes were differentiated as previously described^35,85^, maintained in RPMI (Sigma-Aldrich), B27, and antibiotic–antimycotic (Thermo Fisher), and used at ~ day 30. All hPSC-CMs were cultured at 37ºC in 21% O_2_ and 5% CO_2_.

### Conditioned media production and modification

cMSC were seeded in maintenance medium onto collagen-coated 6-well plates at 20,000–100,000 cells/cm^2^. At 24 h, the medium was aspirated, plates washed, and the respective hPSC-CM medium was added. Conditioned media were collected 20–24 h later, passed through a 0.2 μm filter (GE Healthcare). Conditioned media were injected at the time of coronary artery ligation in vivo and were added to hPSC-CMs at the time of stress induction, unless otherwise indicated. Pierce PES columns with a cutoff of 3 kDa (Thermo Fisher) were used to concentrate the media for in vivo experiments and to discriminate between low and higher molecular weight factors. Thermolability testing was performed by heating at 90 °C for 10 min.

### Myocardial infarction

Coronary artery ligation was performed as described previously^5,23^. Briefly, induction of general anaesthesia was performed with 4% isoflurane, then maintained at 2% in 100% O_2_. Mice received 0.024 mg buprenorphine subcuteneously (i.e. average dose, 1.1 mg/kg; Vetergesic, Alstoe Animal Health, UK), and were intubated and ventilated with a tidal volume of 250 μl and respiratory rate 150 breaths/min (Hugo-Sachs MiniVent type 845; Harvard Apparatus Ltd., Kent, UK). Throughout the surgery heart rate, ECG and core body temperature were constantly monitored. A left thoracotomy was performed in the fourth intercostal space followed by removal of the pericardium. A 6-0 polypropylene suture was used to permanently occlude the left anterior descending (LAD). Myocardial ischemia was confirmed by blanching of the myocardium downstream of the suture and clear ST-segment elevation on ECG tracings. Concentrated conditioned media or vehicle were immediately injected into the infarct border zone (2 injections of 10 μl each). After surgery, mice were allowed to recover in a heated chamber for 20 min, then moved to a normal holding cage with supplemental heat if necessary. Twenty-four hours after MI, the hearts were collected for immunohistochemistry. Briefly, general anaesthesia was induced with 5% isofluorane and maintained at 2.5% with 100% O_2_. Animals were subsequentally perfused via intracardiac injection with PBS for 30 s and 4% PFA in PBS for 60 s prior to heart removal.

### Immunohistochemistry and confocal imaging

Isolated hearts were fixed in 4% PFA solution for 2 h. Subsequently, the organs were incubated in cryoprotective solutions containing gradually higher concentrations of sucrose for the indicted periods of time: 10% (~ 16 h), 20% (8 h) and 30% (~ 16 h) sucrose in distilled H_2_O. Hearts were then frozen in OCT and stored at − 80 °C until sectioning.

For tissue staining, 10 μm sections were washed and permeabilized using 0.5% Tween-20 in PBS. Apoptosis staining was performed using the Click-iT TUNEL Alexa Fluor 594 Imaging Assay (Invitrogen, C10246). After TUNEL staining, sections were blocked in 5% donkey serum and 1% BSA for 30 min, washed twice in PBS for 3 min and incubated with primary antibodies (Table S1 in Supplementary Data) overnight at 4 °C. The following day, sections were washed twice in PBS for 3 min and incubated with secondary antibodies for 2 h at 4 °C. Sections were then washed twice in PBS for 3 min, mounted in Vectashield antifade mounting medium with DAPI (Vector Laboratories), and kept at 4 °C until scanned. Slides were raised to room temperature 15 min prior to image acquisition.

Confocal imaging was performed with a Zeiss LSM-780 inverted microscope, using a EC Plan Neofluor 40×/1.3 oil objective. Images were acquired as a z-stack, ranging from 17–21 optical sections at 1.5 μm intervals, with a pinhole aperture of 1.3 μm. For each heart, we quantified 2 sections per region and 3 regions per mouse encompassing 18–24 fields each, with 10% overlap, for the infarct area, border zone and remote area (interventricular septum). Higher resolution images were acquired as representative images of the TUNEL staining by acquiring z-stacks consisting of 50 optical sections at a 0.36 μm interval, with a pinhole aperture of 1.3 μm and 2 × zoom. Post-acquisition processing was performed using Fiji (ImageJ2). TUNEL staining in cardiomyocytes (an average of ~ 3,400 cardiomyocytes counted per heart) was scored manually by an individual blinded to the experimental conditions.

### Co-culture

HTS Transwell 96-well permeable supports with polyethylene terephthalate membranes and 1.0 micron pores were used (Corning). Cardiomyocytes were seeded onto the bottom well at a density of 100,000 cells/cm^2^ in Cor.4U Complete Culture Medium. One day before starting co-cultures, cMSC were seeded at a density of 100,000 cells/cm^2^ on the apical side of inserts coated with 50 μg/ml collagen in cMSC-mantainance medium. Seven days after human cardiomyocytes’ seeding, inserts with cMSC were added on top of the cardiomyoctes and menadione (Sigma-Aldrich) treatment was started (Fig. 2a). For the control, the stressor was added to cardiomyocytes cultures in the absence of cMSC.

### Cell staining and high-throughput imaging

To monitor cardiomyocyte death, hPSC-CMs were stained with DRAQ7 (Biolegend) at a final concentration of 3 μmol/l and counter-stained with 8 μmol/l Hoechst 33342 (Sigma-Aldrich). Mitochondrial depolarisation was visualised using 20 nmol/l TMRM (Thermo Fisher) for 30 min at 37 °C; 0.01 mmol/l carbonyl cyanide-4-(trifluoromethoxy)phenylhydrazone (FCCP; Abcam), 0.001 mmol/l oligomycin (Abcam) and 0.001 mmol/l rotenone (Abcam), 15 min before scanning, were used as the control for mitochondrial depolarization. CellROX Green (Invitrogen) and MitoSOX Red (Invitrogen) were used to detect cytosolic reactive oxygen species (ROS) and mitochondrial superoxide, after incubation with 5 μmol/l dye at 37 °C for 30 or 10 min, respectively. ROS-Glo H_2_O_2_ assay (Promega) was used to moderate the production of H_2_O_2_ in human IMR-90 after menadione stress. Click-iT EdU HCS assay (Invitrogen) was used to detect cardiomyocytes in S phase for the proliferation assay after incubation with the cells for 24 h and subsequent click-reaction staining. Nuclei were counterstained with Hoecsht 33342 as above.

Live-cell images were acquired at 37 °C with 5% CO_2_ on a Cellomics ArrayScan VTI platform (Thermo Fisher), using the HCS Studio with Cellomics Scan Version 6.4.4 software (Thermo Fisher). The automated Zeiss Observer Z1 epifluorescence microscope was used to acquire 6–20 fields per well (≥ 100 cells per well) with suitable filter sets at 20× magnification (for TMRM and MitoSOX) or 10× magnification (all others). Fluorescence intensity was recorded in channels 1–4, using the filter sets XF93 Hoechst (DAPI), XF93 FITC (GFP), XF32-TRITC sensitive (dsRed) and XF93Cy5 (Alexa-647), respectively. Experiments were performed in triplicate, except where noted. Images were analyzed using the HCS Studio BioApplication Cell Health Profiling V4 (Thermo Fisher).

After live-cell image acquisition, cells were fixed in 3.7% formaldehyde in PBS for 15 min at room temperature and stored at 4 °C. DNA fragmentation was detected using the Click-iT Plus TUNEL Alexa Fluor 594 assay for in situ apoptosis detection (Invitrogen) and Cellomics ArrayScan VTI.

### Bulk RNASeq

RNA isolation from human IMR90-cardiomyocytes cultured at 100,000 cells/cm^2^ in 6-well plates was performed using DirectZol RNA Miniprep kit (Zymo Research). Samples included four groups: (1) untreated cells; (2) cells treated with 20 μM menadione; (3) cells maintained in cMSC-conditioned media and (4) cells treated with menadione and cMSC-conditioned media. Cells were harvested for RNA isolation at 24 h after the treatments were started. Quality control was checked by RNA Qubit and TapeStation before and after library preparation and sequencing (Genewiz, UK). Sequencing was performed using Illumina HiSeq 2 × 150 bp configuration. Raw data were downloaded in FASTQ format for review of the QC results and downstream analysis. Trim Galore (https://www.bioinformatics.babraham.ac.uk/projects/trim_galore/) was used to trim the adapter sequences and reads were aligned to the human genome (GRCh38) using STAR^86^. Data was then imported to SeqMonk (https://www.bioinformatics.babraham.ac.uk/projects/seqmonk/) to quantify, explore and generate heatmaps. Specifically, quantitation was performed using the RNA-seq pipeline, correcting for genomic DNA contamination and merging transcripts isoforms. The DESeq2 R pipeline was used to identify differentially expressed transcripts between the four samples^87^. Data mining was performed using the ToppFun function in the ToppGene Suite tool to identify Gene Ontologies (GO) relevant to the highly significant differentially expressed genes between samples. Data were deposited on SRA public repository with accession number PRJNA629893.

### Exosome isolation and characterization

Exosomes were enriched and depleted from conditioned medium by ultracentrifugation. Medium was centrifuged at 4 °C for 30 min at 2,500×*g* (Thermo Fisher, Heraeus Multifuge 3SR+). The supernatant was centrifuged at 4 °C for 35 min at 4,565×*g*, passed through a 0.2 μm filter, and centrifuged at 4 °C for 2 h at 110,000×*g* in an Optima XPN ultracentrifuge with a SW32 Ti rotor (Beckman Coulter). The cleared supernatant was the exosome-depleted fraction; pellets were resuspended in Cor.4U medium as the exosome-enriched fraction. For exosome identification, 75,000 4 μm aldehyde/sulphate latex beads (Thermo Fisher) were resuspended in 0.025 mol/l MES buffer [2-(N-Morpholino)ethanesulfonic acid; Sigma-Aldrich], coated with 8 μg CD63 (Biolegend) or rat IgG2α (Biolegend), incubated on a rotator for 20 min in a final volume of 50 μl PBS, then incubated for 30 min with 100 mmol/l glycine (Sigma-Aldrich) to occupy unreacted sites. Media were incubated with the antibody-coated beads in a final volume of 250 μl for 15 min. Exosome-bead complexes were stained with 10 μg/ml FITC labelled anti-CD9 (Biolegend), with gentle agitation for 30 min in the dark. Results were visualised using an LSRII flow cytometer (Becton Dickinson) equipped with five lasers^5^, and were analysed using FlowJo (v10, FlowJo).

### Cytokine arrays

High density array membranes (RayBiotech) with 308 mouse proteins were used to screen mouse cMSC-conditioned media for the presence of secreted factors. Basal medium and non-protective TTF-conditioned medium were also tested as controls. Following collection of conditioned media from cells seeded at 20,000/cm^2^, samples were dialysed overnight in PBS to remove small molecular weight molecules including salts and ions. Protein concentration in each sample was determined using the 660 nm assay (ThermoScientific) before and after dialysis and used to calculate the amount of biotin-labeling reagent to add per sample (as per manufacturer’s protocol). Samples were incubated with biotin-labeling reagent at room temperature for 30 min with gentle shaking prior to addition of 5 μl stop solution to prevent further biotin binding. Spin columns and centrifugation were then used to remove unbound biotin and elute the samples. Membranes were first blocked using the provided blocking buffer for 2 h at room temperature with gentle shaking. Each sample was then diluted five-fold in blocking buffer and added onto the blocked membranes for incubation overnight at 4 °C with gentle shaking. Subsequently, the membranes were washed using the solutions provided by the kit and incubated with HRP-conjugated streptavidin for 2 h at room temperature with gentle shaking. After washing, the membranes were incubated with detection buffer for 2 min. Signal was captured on X-ray films (Amersham). Results were analysed using Microsoft Excel and Fiji and Java scripts were used for automation of analysis in ImageJ.

### Flow cytometry and sorting

Flow cytometry and sorting were performed using a FACS Aria II (Becton Dickinson) equipped with 355 nm ultraviolet, 405 nm violet, 488 nm blue, 561 nm yellow–green and 640 nm red lasers and analysed using FlowJo v10. Antibodies and dyes are detailed in Table S1.

### Human subjects

Adult human heart samples were obtained from the NIH-funded National Disease Research Interchange (NDRI), with protocols reviewed and approved annually by the University of Pennsylvania Institutional Review Board. Human cardiac stromal cells were obtained by four rounds of 7 min enzymatic digestions with 90 μg/ml Liberase (Roche) and 50 μg/ml DNase I (Roche), followed by filtering through 70 μm sterile mesh. The cells were then purified by Percoll gradient centrifugation, to remove debris and red blood cells.

### Single cell qRT-PCR

For single cell qRT-PCR single viable cells were sorted directly into 96-well plates containing 10 μl of the reaction mixture for pre-amplification, using CellDirect One-Step qRT–PCR kits (Invitrogen)^5^. Pre-amplification was performed for 22 cycles (Veriti Thermal Cycler, Applied Biosystems, Thermo Fisher). Non-template samples were included in each run at the pre-amplification stage as negative controls. Quantitative amplification was performed using Dynamic Array chips for 96 assays × 96 samples on the BioMark HD system (Fluidigm). For TaqMan primer/probe sets (ABI, Thermo Fisher) see Supplementary Table S1. Ct values were calculated and exported using Fluidigm software (v4; Fluidigm). Data analysis was performed based on cycle threshold (Ct) values obtained by the Fluidigm software, using an R pipeline developed in house. Outliers were identified from quantile–quantile plots of cycle threshold (Ct) values for an internal control gene (Ubc), using a quality control step based on robust standardized expression fractions and expression levels^88^. Ct values were normalised on a per-sample basis, using Ubc as control, expressing all Ct values as their difference to the Ct value of Ubc for the same sample (ΔCt_sample, gene_ = Ct_sample, gene_ − Ct_sample, Ubc_); ΔCt values were subsequently centered on the sample mean (ΔCt_centered_ = ΔCt_sample, gene_ − Ct_sample mean_). Opposite values (− ΔCt_centered_) were used for visualisation and further analyses. Euclidean distances among samples and among genes were used to compute hierarchical clustering, using the complete linkage method. Differential expression was determined using ANOVA with Tukey’s test, using a significance level of p < 0.05. Dimensionality reduction was performed by principal component analysis using FactoMineR^89^. All plotting was performed using R base graphics and ggplot2.

### Statistics

GraphPad Prism (versions 6 and 7) was used to perform statistical analyses. Results are shown as the mean ± SEM (*p* < 0.05). Data were analyzed by two-way ANOVA, using the Bonferroni correction for multiple comparisons and Welch’s t test for pairwise comparisons. The immunohistochemistry data were analysed using an unpaired non-parametric one-tail Mann–Whitney test.

## Supplementary information

Supplementary Information 1.

Supplementary Information 2.
