# Supplementary material for: Human pluripotent stem cell-derived cardiomyocytes as a target platform for paracrine protection by cardiac mesenchymal stromal cells
Source: Sci Rep. 2020 Aug 3;10:13016. doi: 10.1038/s41598-020-69495-w (PMC7400574; doi:10.1038/s41598-020-69495-w)
Supplement: Supplementary file 2 — Supplementary Information 2. [file 41598_2020_69495_MOESM2_ESM.docx]

Human pluripotent stem cell-derived cardiomyocytes as a target platform for paracrine protection by cardiac mesenchymal stromal cells

Chrystalla Constantinou, Antonio M. A. Miranda, Patricia Chaves, Mohamed Bellahcene, Andrea Massaia, Kevin Cheng, Sara Samari, Stephen M. Rothery, Anita M. Chandler, Richard P. Schwarz, Sian E. Harding, Prakash Punjabi, Michael D. Schneider & Michela Noseda

Supplementary Information

Supplementary Figure S1

Supplementary Figure S2

Supplementary Figure S3

Supplementary Figure S4

Supplementary Table S5

Supplementary Figure S6

Supplementary Table S7

Supplementary Figure S8

Supplementary Figure S9

Supplementary Figure S10

Supplementary Figure S11

Supplementary Table S12


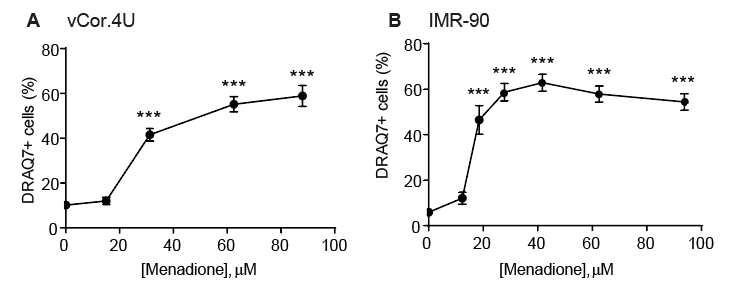
**SUPPLEMENTARY FIGURE S1**

**Supplementary Figure S1. Menadione titration in human PSC-CM lines.** Bar graphs of DRAQ7 uptake after menadione tress in **A**, vCor.4U (n = 3) and **B.** IMR-90 (n = 12). Data are shown as the mean ± SEM. ***, P < 0.0001.

**SUPPLEMENTARY FIGURE S2**


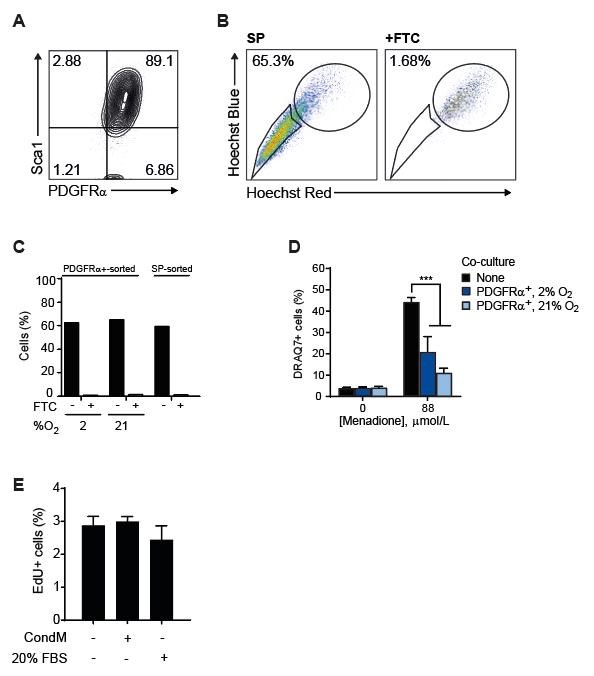


**Supplementary Figure S2. Human cardiomyocyte protection by PDGFRα^+^ SP-enriched mouse cMSCs, expanded without pre-selection for the SP phenotype. A**, Hematopoietic lineage marker-negative (Lin^-^) Sca1^+^ cardiac cells encompass PDGFRα^+^ and SP cells, with a partial overlap between the latter two [^1^](#_ENREF_1). cMSCs used in this study routinely were sorted as Lin^-^ Sca1^+^ SP (“SP-sorted”). As expected, flow cytometry 2D contour plots confirmed the co-expression and enrichment of Sca1 and PDGFRα after in vitro expansion of SP-sorted cardiac cells [^1^](#_ENREF_1). Quadrants indicate the borders of the isotype controls. **B**, As a complementary approach, we also tested the protective effect of Lin^-^, Sca1^+^ CD31^-^ PDGFRα^+^-cardiac cells (PDGFRα-sorted) [^1^](#_ENREF_1). After *in vitro* expansion, enrichment for the SP phenotype was confirmed as predicted. Representative dot plot of SP staining, illustrated for PDGFRα-sorted cardiac cells maintained in 21% O2. FTC, Fumitremorgin C. **C**, Bar graph showing marked enrichment for the SP phenotype in PDGFRα^+^ Sca1^+^ cells expanded in either 21% or 2% O_2_. The prevalence was equal to that in cells derived from homogeneous, flow-sorted SP cells, shown at the right for comparison. Roughly 65% of cells displayed the SP dye-efflux phenotype associated with stem cells, regardless of the culture or cell isolation method used. Data shown are single measurements for the six conditions tested. **D**, Enhanced survival of vCor.4U human ventricular myocytes treated with conditioned media from the PDGFRα-sorted (SP-enriched) cells shown in B and C. Bar graph of DRAQ7 uptake 24 h after oxidative stress. n = 6. Data are shown as the mean ± SEM; ***, P < 0.0001. **E,** Neither treatment with mouse cMSC conditioned media nor supplementation with FBS affect human cardiomyocyte proliferation. Bar graph of EdU staining in v.Cor4U cells 48 h after mouse cMSC-conditioned media treatment or 20% FBS supplementation after incubation with EdU reagent for 24 hours. Data are shown as the mean ± SEM. n = 8 (n = 3 for FBS-treated samples).

**SUPPLEMENTARY FIGURE S3**


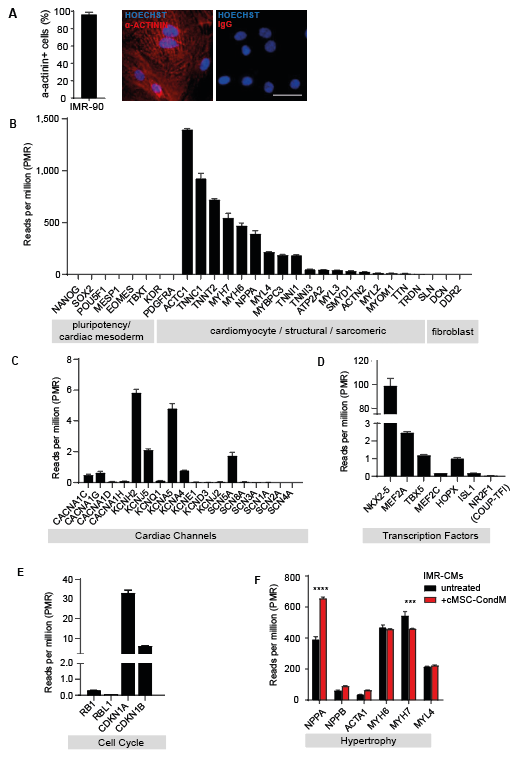


**Supplementary Figure S3: IMR90-cardiomyocyte characterisation.** **A,** Bar graph and representative images for α-actinin staining and the isotype control in cultured IMR90-cardiomyocytes showing high purity. Data are mean ± SEM; n=2. **B,** Bar graphs of reads per million for the genes involved in cardiac development and sarcomere identification; **C,** Cardiac channel expression; **D,** Cardiac transcription factors; **E,** Cell cycle genes **F,** Hypertrophy genes. Reads per million obtained from bulk RNASeq data-set on IMR90 cardiomyocytes at baseline, normalised for transcript length. Data are mean ± SEM; n = 3.

**SUPPLEMENTARY FIGURE S4**


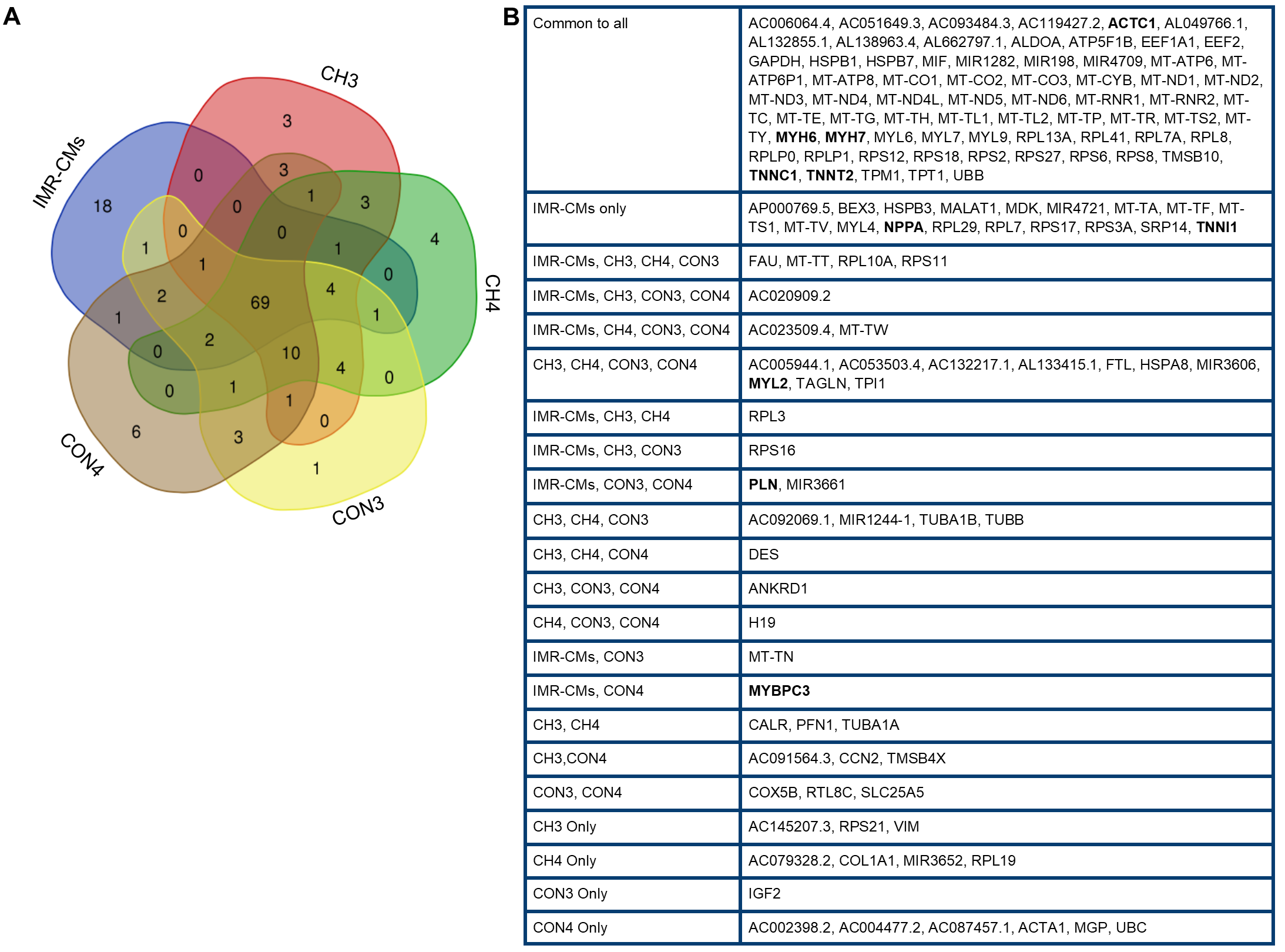


**Supplemenrary Figure S4: IMR90-derived CMs have high expression of several cardiomyocyte markers compared to other hPSC-derived CMs**. **A,** Venn diagram intersecting the top 100 expressed genes in IMR90-derived CMs and published hPSC-derived CMs; **B,** List of genes for each intersection on the Venn diagram, with cardiomyocyte specific genes in bold. IMR90-derived CMs have a higher proportion of cardiomyocyte genes (IMR-CMs - 9; CON3 - 7; CON4 - 8; CH3 - 6; CH4 - 6)

**SUPPLEMENTARY TABLE S5:** Top 100 highly expressed genes in IMR90-CMs and control hPSC-CM lines.

| Rank | IMR-CMs | CH3 | CH4 | CON3 | CON4 | Rank | IMR-CMs | CH3 | CH4 | CON3 | CON4 |
| --- | --- | --- | --- | --- | --- | --- | --- | --- | --- | --- | --- |
| 1 | MT-RNR2 | MYL7 | MT-CO1 | MT-CO2 | MT-CO2 | 51 | TMSB10 | MYH6 | TUBB | TPM1 | ANKRD1 |
| 2 | MT-ATP8 | MT-CO2 | MT-CO2 | MT-CO1 | MT-ATP6 | 52 | RPS2 | AL133415.1 | RPS27 | MT-TR | RPL8 |
| 3 | MT-CO1 | MT-CO3 | MT-ATP8 | MT-ATP8 | MT-ATP8 | 53 | RPLP1 | MIF | RPL41 | ATP5F1B | RPS2 |
| 4 | MT-ATP6 | MT-CO1 | MT-CO3 | MT-ATP6 | MT-CO1 | 54 | HSPB7 | MYH7 | TPM1 | HSPB7 | ATP5F1B |
| 5 | MT-TC | MT-ATP6 | MT-ATP6 | MYL7 | MT-CO3 | 55 | MT-TW | RPL13A | RPLP1 | RPLP1 | TPM1 |
| 6 | MT-TY | MT-RNR2 | MT-ND4 | MT-CO3 | MT-RNR2 | 56 | AC093484.3 | TUBA1B | FTL | RPS27 | UBB |
| 7 | MT-ND4L | MT-ATP8 | MT-RNR2 | MT-RNR2 | MT-ND4 | 57 | RPLP0 | MT-TP | RPL13A | TUBA1B | TNNT2 |
| 8 | MT-CO2 | AC006064.4 | MT-ND4L | MT-ND4 | MYL7 | 58 | RPS16 | RPLP1 | HSPA8 | TUBB | AL662797.1 |
| 9 | MT-ND4 | MT-ND4 | MT-ND6 | MT-ND4L | MT-ND6 | 59 | RPL7A | RPL7A | TUBA1B | RPL13A | MT-TR |
| 10 | MT-CO3 | MT-TY | MT-CYB | MT-ND6 | MT-ND4L | 60 | MT-TR | MIR3606 | TAGLN | RPL7A | RPLP0 |
| 11 | MT-ND6 | MT-TC | MYL7 | MT-CYB | MT-CYB | 61 | BEX3 | MT-RNR1 | TNNT2 | AC053503.4 | H19 |
| 12 | MT-TN | MT-ND6 | MT-ND5 | MT-TE | MT-ND3 | 62 | RPS11 | MIR4709 | AL133415.1 | HSPA8 | RPS27 |
| 13 | MT-CYB | ACTC1 | MT-TE | MT-ND5 | MT-TE | 63 | MYL6 | RPLP0 | UBB | TPT1 | TPT1 |
| 14 | MYL7 | AL132855.1 | MT-ND2 | MT-ND3 | AL132855.1 | 64 | MIR4709 | MT-TR | MT-TP | AC020909.2 | RPL41 |
| 15 | MT-ND5 | MT-TE | MT-ND3 | MT-TY | MT-ND5 | 65 | MYL4 | RPL41 | RPL7A | FTL | RPLP1 |
| 16 | MT-TE | MT-ND3 | AC006064.4 | AC006064.4 | MT-ND2 | 66 | MT-TV | TAGLN | AC092069.1 | TNNT2 | TMSB4X |
| 17 | MT-ND3 | MT-CYB | MT-ND1 | MT-TC | MT-TY | 67 | RPS18 | AC020909.2 | ATP5F1B | RPL41 | AC002398.2 |
| 18 | MT-ND2 | AC051649.3 | MT-TY | AL132855.1 | AC051649.3 | 68 | TPT1 | DES | MIF | AC005944.1 | TAGLN |
| 19 | MT-ND1 | MT-ND4L | AC051649.3 | MT-ND2 | AC006064.4 | 69 | EEF2 | HSPB7 | RPS18 | RPS18 | RPL13A |
| 20 | MT-TS1 | TNNC1 | AL132855.1 | ACTC1 | ACTC1 | 70 | MIF | AC005944.1 | MT-TW | EEF2 | FTL |
| 21 | MT-TA | GAPDH | ACTC1 | MT-ND1 | MT-TC | 71 | AC020909.2 | TUBB | TPT1 | MIR4709 | AC020909.2 |
| 22 | MT-RNR1 | MT-ND1 | MT-TC | AC051649.3 | MT-ND1 | 72 | AL662797.1 | MIR1244-1 | AC079328.2 | AL049766.1 | AC005944.1 |
| 23 | AL132855.1 | MIR1282 | GAPDH | GAPDH | MT-RNR1 | 73 | MIR198 | AC119427.2 | MYL2 | MT-TW | EEF2 |
| 24 | ACTC1 | MT-ND5 | MT-TL1 | MTATP6P1 | AC053503.4 | 74 | MYL9 | TPT1 | H19 | PLN | MIF |
| 25 | MT-TG | MT-ND2 | MTATP6P1 | MT-TH | MTATP6P1 | 75 | MYBPC3 | RPS18 | RPS12 | AC023509.4 | MIR4709 |
| 26 | AC006064.4 | AL138963.4 | MT-TS2 | AC132217.1 | MIR1282 | 76 | RPS8 | AL049766.1 | RPS6 | ALDOA | RPS18 |
| 27 | MT-TP | AC093484.3 | MT-TH | MT-TL1 | GAPDH | 77 | RPS12 | MYL9 | DES | RPS8 | MYL9 |
| 28 | MT-TL1 | TMSB10 | MIR3606 | AL138963.4 | TNNC1 | 78 | PLN | AC132217.1 | MYL9 | MIR3661 | RPL7A |
| 29 | MIR1282 | MT-TS2 | TNNC1 | TNNC1 | AL138963.4 | 79 | UBB | HSPA8 | ALDOA | IGF2 | ALDOA |
| 30 | TNNC1 | EEF1A1 | MIR1282 | MT-TG | MYH7 | 80 | RPL10A | RPS8 | COL1A1 | TPI1 | MT-TW |
| 31 | HSPB1 | MT-TH | AL138963.4 | MT-TS2 | MT-TH | 81 | RPL41 | RPL10A | TPI1 | H19 | HSPA8 |
| 32 | MT-TH | HSPB1 | MT-TG | MIR1282 | MT-TL1 | 82 | ALDOA | ALDOA | MIR4709 | RPS6 | CCN2 |
| 33 | TNNT2 | MYL6 | MT-TL2 | MT-RNR1 | DES | 83 | MALAT1 | TPI1 | RPS8 | AL133415.1 | MGP |
| 34 | EEF1A1 | MTATP6P1 | EEF1A1 | EEF1A1 | MT-TG | 84 | TNNI1 | TMSB4X | EEF2 | MYL2 | AC119427.2 |
| 35 | AL138963.4 | MT-TG | AC132217.1 | AC093484.3 | EEF1A1 | 85 | ATP5F1B | RPS6 | AC023509.4 | ANKRD1 | AC091564.3 |
| 36 | MTATP6P1 | MT-TL2 | AL662797.1 | MYH7 | MT-TS2 | 86 | SRP14 | RPS12 | RPL10A | TAGLN | RTL8C |
| 37 | GAPDH | AC053503.4 | MT-RNR1 | MT-TL2 | AC093484.3 | 87 | MIR3661 | CALR | MT-TT | MT-TN | AL133415.1 |
| 38 | MYH7 | AL662797.1 | MIR198 | MT-TP | MYH6 | 88 | RPL29 | ATP5F1B | AC119427.2 | MYL9 | RPS6 |
| 39 | AC051649.3 | FTL | TMSB10 | AL662797.1 | ACTA1 | 89 | FAU | CCN2 | AC005944.1 | RPL10A | RPS8 |
| 40 | AC119427.2 | MT-TL1 | AC093484.3 | RPS2 | MIR3606 | 90 | RPL3 | FAU | CALR | RPS12 | RPS12 |
| 41 | MYH6 | RPS2 | HSPB1 | HSPB1 | HSPB1 | 91 | MDK | RPS11 | MIR3652 | AC092069.1 | AC023509.4 |
| 42 | AP000769.5 | UBB | MYL6 | MIR198 | MT-TL2 | 92 | HSPB3 | RPS21 | AL049766.1 | AC119427.2 | AC087457.1 |
| 43 | MT-TS2 | RPL8 | MYH7 | MYL6 | MT-TP | 93 | RPL7 | EEF2 | RPS11 | FAU | SLC25A5 |
| 44 | RPL13A | AC092069.1 | RPS2 | MYH6 | HSPB7 | 94 | RPS3A | RPL3 | RPL3 | MT-TT | TPI1 |
| 45 | RPL8 | TPM1 | MT-TR | TMSB10 | MYL2 | 95 | MIR4721 | VIM | TUBA1A | MIR1244-1 | UBC |
| 46 | MT-TT | MYL2 | AC053503.4 | RPL8 | MYL6 | 96 | RPS17 | AC145207.3 | FAU | RPS11 | MYBPC3 |
| 47 | MT-TL2 | TNNT2 | MYH6 | UBB | MIR198 | 97 | AL049766.1 | TUBA1A | MIR1244-1 | RTL8C | AC004477.2 |
| 48 | NPPA | RPS27 | RPLP0 | RPLP0 | PLN | 98 | MT-TF | AC091564.3 | PFN1 | SLC25A5 | COX5B |
| 49 | TPM1 | ANKRD1 | RPL8 | MIR3606 | AC132217.1 | 99 | RPS6 | PFN1 | RPS16 | COX5B | MIR3661 |
| 50 | RPS27 | MIR198 | HSPB7 | MIF | TMSB10 | 100 | AC023509.4 | MT-TT | RPL19 | RPS16 | AL049766.1 |


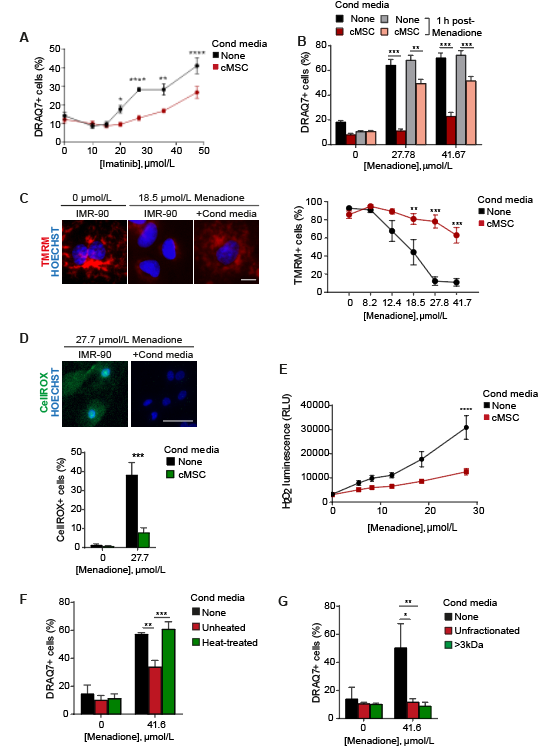
**SUPPLEMENTARY FIGURE S6**

**Supplementary Figure S6. Paracrine protection of IMR-90 hPSC-CMs by mouse cMSC-conditioned media. A**, Line graph of DRAQ7 uptake, showing significant protection of human IMR-90 by mouse cMSC-conditioned medium following Imatinib stress. n = 8. B, Bar graph of DRAQ7 uptake, showing significant protection even when conditioned medium is given 1 h after menadione. n = 11. **C**, Representative images and line graph of mitochondrial TMRM, 24 h after oxidative stress ± cMSC-conditioned media. n = 9. **D,** Representative images and bar graph of CellROX staining 8 h after menadione ± cMSC-conditioned media. n = 12. **E,** Line graph of ROS-GLO H_2_O_2_ luminescence assay, 24 h after menadione stress ± cMSC-conditioned media. n = 9. Scale bar, 50 μm. **F**, Protection is mediated by heat-labile factors. n = 6. **G**, The media fraction containing molecules >3 kDa in size mediates the protective effects. n = 3. See Fig. 4 for details. Data are shown as the mean ± SEM. *, P < 0.05; **, P < 0.001; ***, P < 0.0001.

**SUPPLEMENTARY TABLE S7:** Top 50 Gene Ontologies for Biological Processes associated to each combination of samples as denoted per table, obtained using the ToppFun function in ToppGene Suite. Genes used to generate the GOs were filtered using a threshold of log_2_ fold change > 2 and p-value < 0.05. Extended tables including gene IDs per GO can be found in a separate supplementary information file.

| Upregulated in IMR90-CMs by menadione treatment  TOP 50 GO FOR BIOLOGICAL PROCESSES | | | |
| --- | --- | --- | --- |
| ID | Name | q-value FDR B&H | Hit Count in Query List |
|  |  |  |  |
| GO:0042127 | regulation of cell population proliferation | 9.91E-20 | 166 |
| GO:0034097 | response to cytokine | 9.66E-19 | 126 |
| GO:0051241 | negative regulation of multicellular organismal process | 3.95E-18 | 137 |
| GO:0071345 | cellular response to cytokine stimulus | 3.95E-18 | 118 |
| GO:0001775 | cell activation | 3.10E-17 | 138 |
| GO:0050865 | regulation of cell activation | 6.82E-15 | 79 |
| GO:0016477 | cell migration | 7.73E-15 | 143 |
| GO:0001816 | cytokine production | 2.18E-14 | 90 |
| GO:0010941 | regulation of cell death | 2.25E-14 | 153 |
| GO:0001817 | regulation of cytokine production | 2.88E-14 | 84 |
| GO:0043067 | regulation of programmed cell death | 6.74E-14 | 143 |
| GO:0002694 | regulation of leukocyte activation | 8.30E-14 | 73 |
| GO:0008284 | positive regulation of cell population proliferation | 8.30E-14 | 102 |
| GO:0042981 | regulation of apoptotic process | 1.53E-13 | 140 |
| GO:0051094 | positive regulation of developmental process | 1.53E-13 | 132 |
| GO:0022610 | biological adhesion | 2.29E-13 | 125 |
| GO:0019221 | cytokine-mediated signaling pathway | 3.18E-13 | 83 |
| GO:0051674 | localization of cell | 3.18E-13 | 147 |
| GO:0048870 | cell motility | 3.18E-13 | 147 |
| GO:0007155 | cell adhesion | 3.18E-13 | 124 |
| GO:0001568 | blood vessel development | 3.47E-13 | 85 |
| GO:0002684 | positive regulation of immune system process | 4.00E-13 | 110 |
| GO:0033993 | response to lipid | 4.25E-13 | 94 |
| GO:0030155 | regulation of cell adhesion | 4.82E-13 | 80 |
| GO:0001525 | angiogenesis | 6.68E-13 | 71 |
| GO:0045321 | leukocyte activation | 6.68E-13 | 116 |
| GO:0048514 | blood vessel morphogenesis | 8.28E-13 | 78 |
| GO:0002682 | regulation of immune system process | 8.90E-13 | 137 |
| GO:0001944 | vasculature development | 9.08E-13 | 86 |
| GO:0072358 | cardiovascular system development | 2.05E-12 | 86 |
| GO:0045597 | positive regulation of cell differentiation | 3.85E-12 | 102 |
| GO:0035239 | tube morphogenesis | 4.90E-12 | 94 |
| GO:1901700 | response to oxygen-containing compound | 5.22E-12 | 138 |
| GO:0006954 | inflammatory response | 7.63E-12 | 79 |
| GO:0022407 | regulation of cell-cell adhesion | 1.12E-11 | 55 |
| GO:0098609 | cell-cell adhesion | 1.16E-11 | 85 |
| GO:0035295 | tube development | 1.48E-11 | 106 |
| GO:0046649 | lymphocyte activation | 1.51E-11 | 79 |
| GO:0030334 | regulation of cell migration | 1.59E-11 | 93 |
| GO:0051093 | negative regulation of developmental process | 1.88E-11 | 101 |
| GO:0002683 | negative regulation of immune system process | 1.93E-11 | 59 |
| GO:1903037 | regulation of leukocyte cell-cell adhesion | 1.93E-11 | 46 |
| GO:0002521 | leukocyte differentiation | 2.22E-11 | 64 |
| GO:0051249 | regulation of lymphocyte activation | 3.66E-11 | 60 |
| GO:0002696 | positive regulation of leukocyte activation | 4.16E-11 | 52 |
| GO:0009611 | response to wounding | 4.16E-11 | 74 |
| GO:0007159 | leukocyte cell-cell adhesion | 4.74E-11 | 48 |
| GO:0050867 | positive regulation of cell activation | 5.65E-11 | 53 |
| GO:1903039 | positive regulation of leukocyte cell-cell adhesion | 6.10E-11 | 38 |
| GO:0048646 | anatomical structure formation involved in morphogenesis | 6.10E-11 | 109 |

| Downregulated in IMR90-CMs by menadione treatment  TOP 50 GO FOR BIOLOGICAL PROCESSES | | | |
| --- | --- | --- | --- |
| ID | Name | q-value FDR B&H | Hit Count in Query List |
| GO:0060047 | heart contraction | 5.66E-08 | 26 |
| GO:0003015 | heart process | 5.66E-08 | 26 |
| GO:0044057 | regulation of system process | 1.47E-07 | 39 |
| GO:0030001 | metal ion transport | 5.82E-07 | 46 |
| GO:1903522 | regulation of blood circulation | 5.82E-07 | 25 |
| GO:0006936 | muscle contraction | 1.24E-06 | 26 |
| GO:0008015 | blood circulation | 1.90E-06 | 33 |
| GO:0003012 | muscle system process | 1.97E-06 | 30 |
| GO:0003013 | circulatory system process | 2.11E-06 | 33 |
| GO:0070252 | actin-mediated cell contraction | 2.44E-06 | 15 |
| GO:0035637 | multicellular organismal signaling | 2.45E-06 | 19 |
| GO:0008016 | regulation of heart contraction | 2.45E-06 | 21 |
| GO:0043269 | regulation of ion transport | 2.45E-06 | 38 |
| GO:0030048 | actin filament-based movement | 2.45E-06 | 16 |
| GO:0034762 | regulation of transmembrane transport | 2.45E-06 | 33 |
| GO:0098662 | inorganic cation transmembrane transport | 6.00E-06 | 42 |
| GO:0006941 | striated muscle contraction | 6.00E-06 | 17 |
| GO:0034765 | regulation of ion transmembrane transport | 6.61E-06 | 29 |
| GO:0060048 | cardiac muscle contraction | 7.30E-06 | 15 |
| GO:0006813 | potassium ion transport | 7.64E-06 | 20 |
| GO:0098660 | inorganic ion transmembrane transport | 7.74E-06 | 44 |
| GO:0007267 | cell-cell signaling | 1.18E-05 | 63 |
| GO:0006812 | cation transport | 2.43E-05 | 51 |
| GO:0098655 | cation transmembrane transport | 2.43E-05 | 44 |
| GO:0034220 | ion transmembrane transport | 2.46E-05 | 52 |
| GO:0071805 | potassium ion transmembrane transport | 2.46E-05 | 18 |
| GO:0061337 | cardiac conduction | 2.51E-05 | 14 |
| GO:0006811 | ion transport | 2.70E-05 | 63 |
| GO:0098916 | anterograde trans-synaptic signaling | 3.80E-05 | 37 |
| GO:0007268 | chemical synaptic transmission | 3.80E-05 | 37 |
| GO:0099537 | trans-synaptic signaling | 4.71E-05 | 37 |
| GO:0002027 | regulation of heart rate | 5.04E-05 | 12 |
| GO:0055085 | transmembrane transport | 5.92E-05 | 59 |
| GO:0099536 | synaptic signaling | 6.08E-05 | 37 |
| GO:0001508 | action potential | 8.66E-05 | 15 |
| GO:0007610 | behavior | 8.75E-05 | 32 |
| GO:0086003 | cardiac muscle cell contraction | 9.04E-05 | 10 |
| GO:1990573 | potassium ion import across plasma membrane | 1.43E-04 | 8 |
| GO:0086012 | membrane depolarization during cardiac muscle cell action potential | 1.69E-04 | 6 |
| GO:0051480 | regulation of cytosolic calcium ion concentration | 1.75E-04 | 22 |
| GO:0042391 | regulation of membrane potential | 1.92E-04 | 25 |
| GO:0086091 | regulation of heart rate by cardiac conduction | 2.61E-04 | 7 |
| GO:0015672 | monovalent inorganic cation transport | 3.03E-04 | 30 |
| GO:0050801 | ion homeostasis | 3.10E-04 | 35 |
| GO:0086010 | membrane depolarization during action potential | 3.49E-04 | 7 |
| GO:0086001 | cardiac muscle cell action potential | 3.68E-04 | 9 |
| GO:0055074 | calcium ion homeostasis | 3.74E-04 | 25 |
| GO:0043270 | positive regulation of ion transport | 4.62E-04 | 19 |
| GO:0072507 | divalent inorganic cation homeostasis | 4.62E-04 | 26 |
| GO:0086018 | SA node cell to atrial cardiac muscle cell signaling | 7.22E-04 | 4 |

| Upregulated by cMSC-conditioned media protected vs menadione-stressed IMR90-CMs  TOP 50 GO FOR BIOLOGICAL PROCESSES | | | |
| --- | --- | --- | --- |
| ID | Name | q-value FDR B&H | Hit Count in Query List |
| GO:0006936 | muscle contraction | 4.92E-05 | 20 |
| GO:0008015 | blood circulation | 4.92E-05 | 25 |
| GO:0003013 | circulatory system process | 4.92E-05 | 25 |
| GO:0030001 | metal ion transport | 4.92E-05 | 33 |
| GO:1903522 | regulation of blood circulation | 5.99E-05 | 18 |
| GO:0050801 | ion homeostasis | 8.85E-05 | 30 |
| GO:0086091 | regulation of heart rate by cardiac conduction | 1.05E-04 | 7 |
| GO:0003012 | muscle system process | 2.56E-04 | 21 |
| GO:0002027 | regulation of heart rate | 2.56E-04 | 10 |
| GO:0044057 | regulation of system process | 3.01E-04 | 25 |
| GO:0060048 | cardiac muscle contraction | 3.83E-04 | 11 |
| GO:0006811 | ion transport | 3.83E-04 | 46 |
| GO:0007267 | cell-cell signaling | 3.86E-04 | 45 |
| GO:0035637 | multicellular organismal signaling | 4.33E-04 | 13 |
| GO:0086018 | SA node cell to atrial cardiac muscle cell signaling | 4.33E-04 | 4 |
| GO:0086015 | SA node cell action potential | 4.33E-04 | 4 |
| GO:0086001 | cardiac muscle cell action potential | 4.33E-04 | 8 |
| GO:0006941 | striated muscle contraction | 4.33E-04 | 12 |
| GO:0048878 | chemical homeostasis | 5.09E-04 | 35 |
| GO:0060047 | heart contraction | 5.09E-04 | 15 |
| GO:0086002 | cardiac muscle cell action potential involved in contraction | 5.18E-04 | 7 |
| GO:0086070 | SA node cell to atrial cardiac muscle cell communication | 5.18E-04 | 4 |
| GO:0098771 | inorganic ion homeostasis | 5.18E-04 | 26 |
| GO:0086003 | cardiac muscle cell contraction | 5.18E-04 | 8 |
| GO:0034220 | ion transmembrane transport | 5.20E-04 | 37 |
| GO:0003015 | heart process | 5.54E-04 | 15 |
| GO:0006812 | cation transport | 6.02E-04 | 36 |
| GO:0086010 | membrane depolarization during action potential | 7.09E-04 | 6 |
| GO:0007626 | locomotory behavior | 8.30E-04 | 13 |
| GO:0061337 | cardiac conduction | 8.41E-04 | 10 |
| GO:0042391 | regulation of membrane potential | 8.52E-04 | 19 |
| GO:0055080 | cation homeostasis | 8.81E-04 | 25 |
| GO:0098662 | inorganic cation transmembrane transport | 9.09E-04 | 28 |
| GO:0001508 | action potential | 1.27E-03 | 11 |
| GO:0030048 | actin filament-based movement | 1.36E-03 | 10 |
| GO:0086046 | membrane depolarization during SA node cell action potential | 1.39E-03 | 3 |
| GO:0098655 | cation transmembrane transport | 1.39E-03 | 30 |
| GO:0006873 | cellular ion homeostasis | 1.39E-03 | 23 |
| GO:0098660 | inorganic ion transmembrane transport | 1.51E-03 | 29 |
| GO:0055085 | transmembrane transport | 1.54E-03 | 41 |
| GO:0008016 | regulation of heart contraction | 1.54E-03 | 13 |
| GO:1990573 | potassium ion import across plasma membrane | 1.58E-03 | 6 |
| GO:0055082 | cellular chemical homeostasis | 1.62E-03 | 26 |
| GO:0051480 | regulation of cytosolic calcium ion concentration | 1.69E-03 | 16 |
| GO:0070252 | actin-mediated cell contraction | 1.69E-03 | 9 |
| GO:0042310 | vasoconstriction | 2.10E-03 | 8 |
| GO:0086019 | cell-cell signaling involved in cardiac conduction | 2.10E-03 | 5 |
| GO:0098916 | anterograde trans-synaptic signaling | 2.10E-03 | 25 |
| GO:0007268 | chemical synaptic transmission | 2.10E-03 | 25 |
| GO:0030003 | cellular cation homeostasis | 2.38E-03 | 22 |

| Downregulated by cMSC-conditioned media protected vs menadione-stressed IMR90-CMs  TOP 50 GO FOR BIOLOGICAL PROCESSES | | | |
| --- | --- | --- | --- |
| ID | Name | q-value FDR B&H | Hit Count in Query List |
| GO:0042127 | regulation of cell population proliferation | 2.38E-16 | 150 |
| GO:0016477 | cell migration | 1.29E-15 | 139 |
| GO:0010941 | regulation of cell death | 8.48E-15 | 147 |
| GO:0051674 | localization of cell | 8.48E-15 | 145 |
| GO:0048870 | cell motility | 8.48E-15 | 145 |
| GO:2000145 | regulation of cell motility | 1.07E-13 | 99 |
| GO:0030334 | regulation of cell migration | 1.07E-13 | 95 |
| GO:0001525 | angiogenesis | 1.54E-13 | 70 |
| GO:0043067 | regulation of programmed cell death | 8.00E-13 | 133 |
| GO:0001568 | blood vessel development | 8.00E-13 | 81 |
| GO:0001944 | vasculature development | 8.00E-13 | 83 |
| GO:0030335 | positive regulation of cell migration | 1.56E-12 | 66 |
| GO:0072358 | cardiovascular system development | 1.56E-12 | 83 |
| GO:0035295 | tube development | 1.70E-12 | 104 |
| GO:0040012 | regulation of locomotion | 1.70E-12 | 100 |
| GO:0035239 | tube morphogenesis | 2.07E-12 | 91 |
| GO:0048514 | blood vessel morphogenesis | 2.26E-12 | 74 |
| GO:2000147 | positive regulation of cell motility | 2.58E-12 | 67 |
| GO:0048646 | anatomical structure formation involved in morphogenesis | 2.60E-12 | 108 |
| GO:0051241 | negative regulation of multicellular organismal process | 2.62E-12 | 115 |
| GO:0051270 | regulation of cellular component movement | 2.83E-12 | 100 |
| GO:0051272 | positive regulation of cellular component movement | 3.84E-12 | 68 |
| GO:0042981 | regulation of apoptotic process | 6.33E-12 | 128 |
| GO:0008284 | positive regulation of cell population proliferation | 8.77E-12 | 92 |
| GO:0034097 | response to cytokine | 1.13E-11 | 102 |
| GO:0040017 | positive regulation of locomotion | 2.00E-11 | 67 |
| GO:0000165 | MAPK cascade | 2.98E-11 | 88 |
| GO:0023014 | signal transduction by protein phosphorylation | 5.47E-11 | 88 |
| GO:0001816 | cytokine production | 1.06E-10 | 77 |
| GO:0051094 | positive regulation of developmental process | 1.08E-10 | 117 |
| GO:0071345 | cellular response to cytokine stimulus | 1.16E-10 | 94 |
| GO:0072359 | circulatory system development | 1.22E-10 | 101 |
| GO:0001775 | cell activation | 1.25E-10 | 113 |
| GO:0033993 | response to lipid | 2.25E-10 | 83 |
| GO:0022610 | biological adhesion | 2.37E-10 | 110 |
| GO:1902533 | positive regulation of intracellular signal transduction | 3.38E-10 | 89 |
| GO:0007155 | cell adhesion | 3.84E-10 | 109 |
| GO:0009611 | response to wounding | 7.84E-10 | 68 |
| GO:1901700 | response to oxygen-containing compound | 9.77E-10 | 124 |
| GO:0001817 | regulation of cytokine production | 1.86E-09 | 69 |
| GO:0009967 | positive regulation of signal transduction | 2.36E-09 | 122 |
| GO:0042060 | wound healing | 5.22E-09 | 58 |
| GO:0098609 | cell-cell adhesion | 9.59E-09 | 74 |
| GO:0048585 | negative regulation of response to stimulus | 9.59E-09 | 125 |
| GO:0043408 | regulation of MAPK cascade | 9.72E-09 | 71 |
| GO:0008285 | negative regulation of cell population proliferation | 1.06E-08 | 71 |
| GO:0001667 | ameboidal-type cell migration | 1.41E-08 | 50 |
| GO:0070371 | ERK1 and ERK2 cascade | 1.57E-08 | 41 |
| GO:0050865 | regulation of cell activation | 1.68E-08 | 61 |
| GO:0007267 | cell-cell signaling | 2.70E-08 | 122 |

| Upregulated by cMSC-conditioned media protected cells vs untreated IMR90-CMs  TOP 50 GO FOR BIOLOGICAL PROCESSES | | | |
| --- | --- | --- | --- |
| ID | Name | q-value FDR B&H | Hit Count in Query List |
| GO:0071395 | cellular response to jasmonic acid stimulus | 2.20E-05 | 1.81E-04 |
| GO:0009753 | response to jasmonic acid | 2.20E-05 | 1.81E-04 |
| GO:0044598 | doxorubicin metabolic process | 1.66E-04 | 1.37E-03 |
| GO:0030638 | polyketide metabolic process | 1.66E-04 | 1.37E-03 |
| GO:0044597 | daunorubicin metabolic process | 1.66E-04 | 1.37E-03 |
| GO:0042180 | cellular ketone metabolic process | 1.66E-04 | 1.37E-03 |
| GO:0030647 | aminoglycoside antibiotic metabolic process | 1.87E-04 | 1.54E-03 |
| GO:0010876 | lipid localization | 2.58E-04 | 2.12E-03 |
| GO:0006692 | prostanoid metabolic process | 3.31E-04 | 2.73E-03 |
| GO:0006693 | prostaglandin metabolic process | 3.31E-04 | 2.73E-03 |
| GO:0006690 | icosanoid metabolic process | 4.64E-04 | 3.82E-03 |
| GO:0008202 | steroid metabolic process | 5.72E-04 | 4.70E-03 |
| GO:0030595 | leukocyte chemotaxis | 6.72E-04 | 5.53E-03 |
| GO:0042448 | progesterone metabolic process | 6.96E-04 | 5.73E-03 |
| GO:1902644 | tertiary alcohol metabolic process | 6.96E-04 | 5.73E-03 |
| GO:0042445 | hormone metabolic process | 7.94E-04 | 6.53E-03 |
| GO:0006869 | lipid transport | 7.94E-04 | 6.53E-03 |
| GO:0016137 | glycoside metabolic process | 7.94E-04 | 6.53E-03 |
| GO:1901568 | fatty acid derivative metabolic process | 1.61E-03 | 1.32E-02 |
| GO:0060326 | cell chemotaxis | 2.29E-03 | 1.88E-02 |
| GO:1901661 | quinone metabolic process | 2.29E-03 | 1.88E-02 |
| GO:0046457 | prostanoid biosynthetic process | 2.29E-03 | 1.88E-02 |
| GO:0001516 | prostaglandin biosynthetic process | 2.29E-03 | 1.88E-02 |
| GO:0010565 | regulation of cellular ketone metabolic process | 2.30E-03 | 1.89E-02 |
| GO:0097306 | cellular response to alcohol | 2.40E-03 | 1.98E-02 |
| GO:0071799 | cellular response to prostaglandin D stimulus | 2.40E-03 | 1.98E-02 |
| GO:0071798 | response to prostaglandin D | 2.40E-03 | 1.98E-02 |
| GO:0006629 | lipid metabolic process | 2.48E-03 | 2.04E-02 |
| GO:0050900 | leukocyte migration | 2.74E-03 | 2.25E-02 |
| GO:0055114 | oxidation-reduction process | 2.88E-03 | 2.37E-02 |
| GO:0097164 | ammonium ion metabolic process | 2.89E-03 | 2.38E-02 |
| GO:0015711 | organic anion transport | 2.89E-03 | 2.38E-02 |
| GO:0046942 | carboxylic acid transport | 2.95E-03 | 2.43E-02 |
| GO:0015849 | organic acid transport | 2.95E-03 | 2.43E-02 |
| GO:0030593 | neutrophil chemotaxis | 2.98E-03 | 2.46E-02 |
| GO:0033559 | unsaturated fatty acid metabolic process | 3.00E-03 | 2.47E-02 |
| GO:0043068 | positive regulation of programmed cell death | 3.21E-03 | 2.64E-02 |
| GO:0097529 | myeloid leukocyte migration | 3.21E-03 | 2.64E-02 |
| GO:0007584 | response to nutrient | 3.45E-03 | 2.84E-02 |
| GO:1901701 | cellular response to oxygen-containing compound | 3.45E-03 | 2.84E-02 |
| GO:0008207 | C21-steroid hormone metabolic process | 3.61E-03 | 2.97E-02 |
| GO:0006631 | fatty acid metabolic process | 3.66E-03 | 3.01E-02 |
| GO:0042127 | regulation of cell population proliferation | 3.77E-03 | 3.10E-02 |
| GO:0051674 | localization of cell | 3.81E-03 | 3.13E-02 |
| GO:0048870 | cell motility | 3.81E-03 | 3.13E-02 |
| GO:0031394 | positive regulation of prostaglandin biosynthetic process | 3.85E-03 | 3.17E-02 |
| GO:0031622 | positive regulation of fever generation | 3.85E-03 | 3.17E-02 |
| GO:0097305 | response to alcohol | 3.94E-03 | 3.24E-02 |
| GO:0006811 | ion transport | 3.94E-03 | 3.24E-02 |
| GO:1990266 | neutrophil migration | 3.99E-03 | 3.28E-02 |

**SUPPLEMENTARY FIGURE S8**


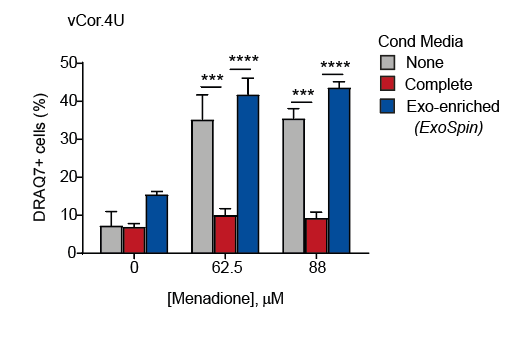


**Supplementary Figure S8. Exosome enriched fraction of mouse cMSC-conditioned media did not protect human vCor4U cardiomyocytes from menadione-induced death.** Bar graph of DRAQ7 uptake in hPSC-CM following incubation with menadione for 24-hours and treatment with complete cMSC-conditioned medium or the fraction enriched in exosomes after purification using the ExoSpin columns. n = 3. Data are shown as the mean ± SEM; ***, P < 0.001; ****, P < 0.0001.


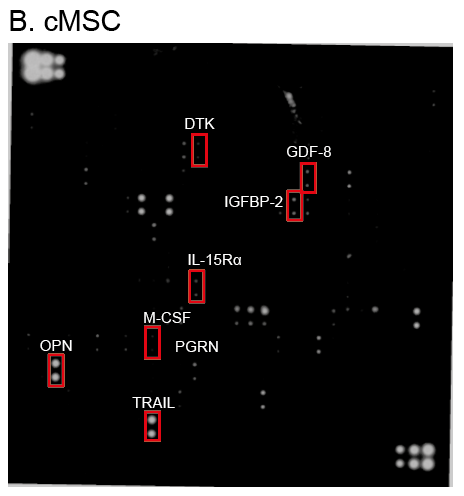

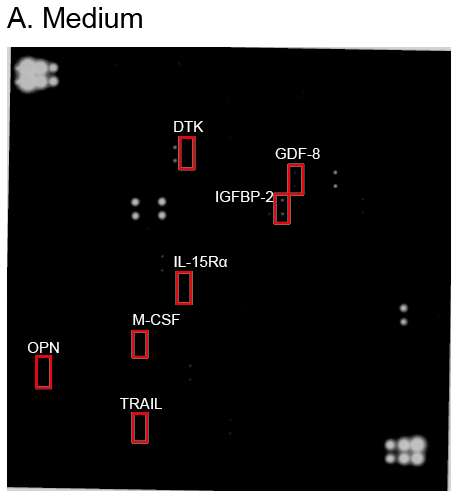
**SUPPLEMENTARY FIGURE S9**

**Supplementary Figure S9. Cytokine array membranes. A,** RPMI base medium; **B,** mouse cMSC-conditioned medium; **C,** mouse TTF-conditioned medium
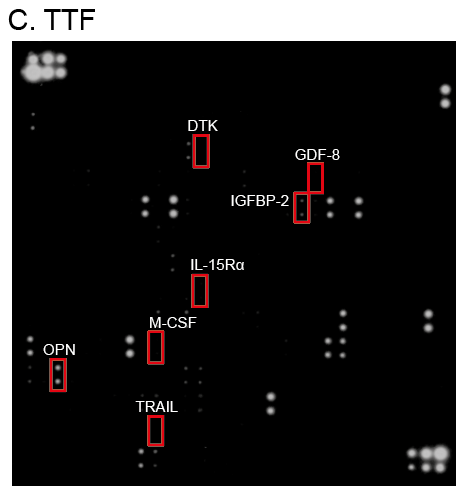
. Factors highlighted in red and labelled are the ones significantly enriched in cMSC-conditioned medium relative to the other two samples. Unpaired t-test, p < 0.05.

##
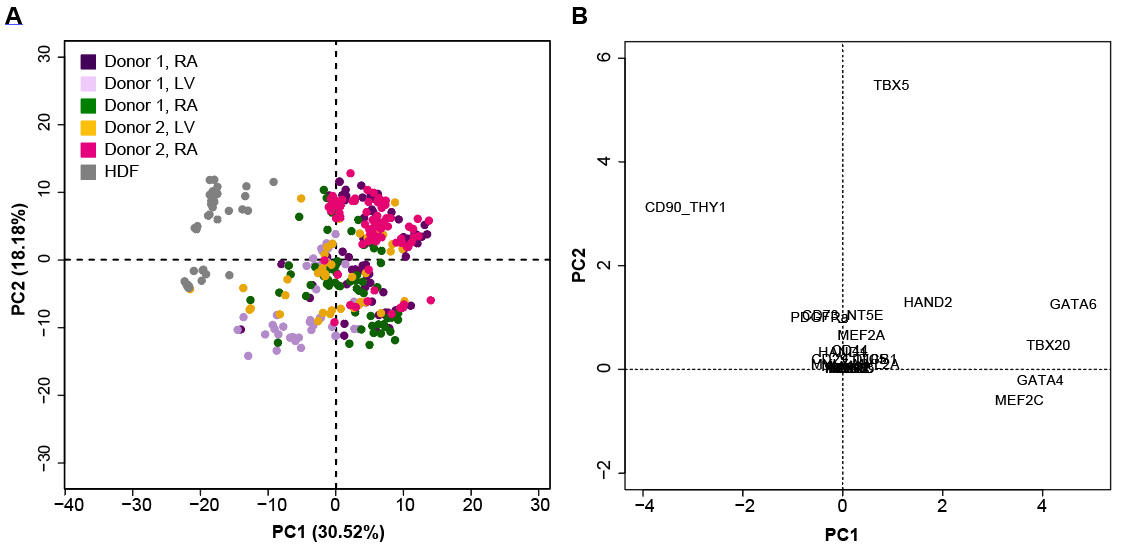
SUPPLEMENTARY FIGURE S10

## Supplementary Figure S10. Human cardiac stromal cells are distinct in molecular signature from human skin fibroblasts. Principal Component Analysis (PCA) of single-cell qRT-PCR profiles from five cardiac samples (comprising two donors and three regions) versus Human Dermal Fibroblasts (HDF). A, PC1 alone (30.52% variability) separates HDF from all five cardiac samples, whereas PC2 (18.18% variability) establishes a distinct separation between different sets of human cardiac stromal cells and a small subset of HDF. B, Gene loadings contributing to each variability indicate that *CD90/THY1* and cardiogenic transcription factors *GATA4*, *GATA6*, *HAND2*, *MEF2C*, *TBX20* explain the cross-group variability captured by PC1, whereas *TBX5* contributes to the separation in PC2. Right Atrium (RA), Right Ventricle (RV) and Left Ventricle (LV).


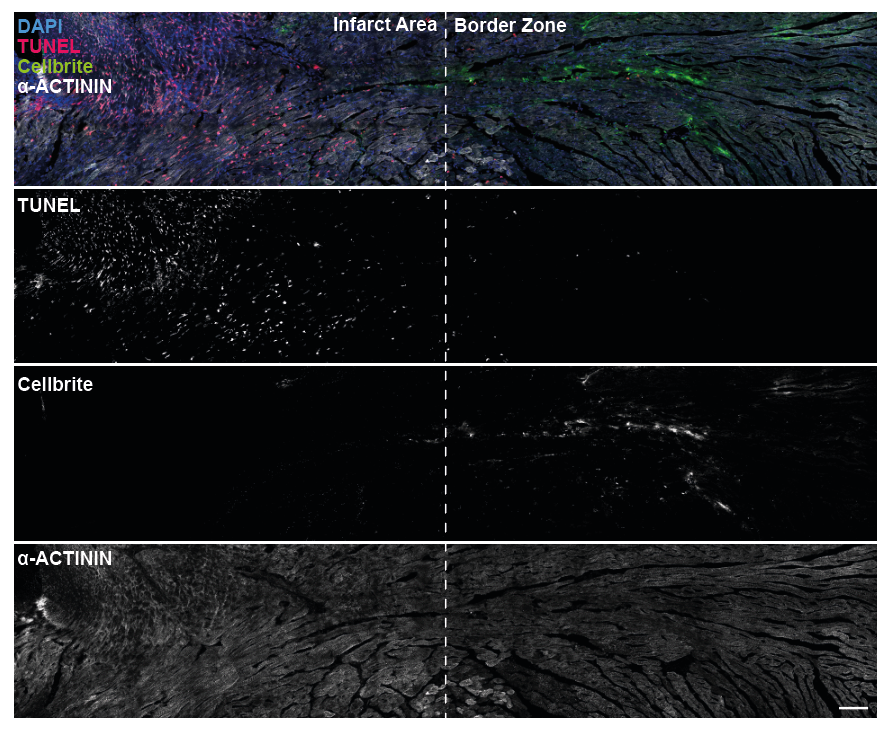
**SUPPLEMENTARY FIGURE S11**

**Supplementary Figure S11. Monitoring of media’s intramyocardial delivery by co-injection with a lipophilic fluorescent carbocyanine dye.** Representative montage compiling 48 tiled fields per row, including the infarct area and contiguous border zone of a heart injected with conditioned medium. **Top row**, Merged image of all four channels. Nuclei are counter-stained with DAPI. **Row 2,** TUNEL assay, to identify apoptotic cells. **Row 3**, Lipophilic fluorescent carbocyanine dye, Cellbrite, to mark the site and track of injection. **Row 4**, Sarcormeric α-actinin staining, to identify cardiomyocytes. Scale bar, 100 μm.

**SUPPLEMENTARY TABLE S12:** Reagents and resources used throughout the manuscript.

| REAGENT or RESOURCE | **SOURCE** | **IDENTIFIER** |
| --- | --- | --- |
| Antibodies | | |
| Anti-human Lineage cocktail, biotin-conjugated | Miltenyi Biotec | 130092211 |
| Anti-mouse Lineage cocktail, biotin-conjugated | Miltenyi Biotec | 130090858 |
| Anti-rabbit α-actinin | Abcam | AB18051 |
| Goat anti-rabbit IgG, PE-conjugated | Cell Signaling | 14705 |
| Mouse anti-human CD105, BV421-conjugated, clone 43A3 | BioLegend | 323219 |
| Mouse anti-human CD29, AF700 conjugated, clone TS2/16 | BioLegend | 303020 |
| Mouse anti-human CD31 (PECAM-1), AF488-conjugated, clone WM59 | BioLegend | 303110 |
| Mouse anti-human CD73, PerCP/Cy5.5-conjugated, clone IM7 | BioLegend | 344014 |
| Mouse anti-human CD90, BV510-conjugated, clone 5E10 | BioLegend | 328125 |
| Rabbit anti-human CD140 (PDGFRα), clone D13C6 | Cell Signalling | 5241 |
| Rat anti-mouse CD140 (PDGFRα), APC-conjugated, clone APA5 | BioLegend | 135908 |
| Rat anti-mouse CD63, clone NVG-2 | BioLegend | 143901 |
| Rat anti-mouse CD9, FITC-conjugated, clone MZ3 | BioLegend | 124807 |
| Rat anti-mouse Ly6A/E (Sca-1), PE-conjugated, clone D7 | BioLegend | 108108 |
| Rat anti-mouse/human CD44, AF647-conjugated, clone IM7 | BioLegend | 103018 |
| Rat IgG2a, κ isotype control antibody, FITC-conjugated, clone RTK2758 | BioLegend | 400505 |
| Rat IgG2a, κ isotype control antibody, clone RTK2758 | BioLegend | 400501 |
| Streptavidin, PE/Cy7-conjugated | BioLegend | 405206 |
| Vectashield Mounting medium with DAPI | Vector | H-1200 |
| Biological Samples | | |
| Human heart tissue | NDRI | N/A |
| Chemicals, Peptides, and Recombinant Proteins | | |
| Aldehyde/sulphate latex beads | Thermo Fisher | A37304 |
| Antibiotic-Antimycotic | Invitrogen | 15240-096 |
| Bovine growth serum | Hyclone | SH30541 |
| B27 | Invitrogen | 17504-044 |
| Buprenorphine | Alstoe Animal Health | - |
| Cardiotrophin-1 | Cell Sciences | CRC700B |
| CellBrite Green cytoplasmic membrane dye | Cambridge Bioscience | BT30021 |
| Cell dissociation buffer, enzyme-free, PBS | Gibco | 13151014 |
| Collagen type I | BD Bioscience | 354236 |
| Cor.4U Culture Medium | Ncardia (Axiogenesis) | Ax-M-HC250 |
| DNAse I | Roche | 11284932001 |
| DRAQ7 | Biolegend | 424001 |
| Dulbecco’s Modified Eagle’s Medium (DMEM) | Invitrogen | 11965-092 |
| FCCP (carbonyl cyanide-4-phenylhydrazone) | Abcam | CAS 370-86-5 |
| Fetal bovine serum | Gibco | 10270 |
| Fibroblast basal medium 2 | PromoCell | C-23220 |
| Fibroblast growth medium 2 supplemental pack | PromoCell | C-39320 |
| Fibronectin, bovine plasma | Sigma-Aldrich | F1141 |
| Formaldehyde solution, neutral buffered, 10% | Sigma | 111M4387 |
| Galactose | Sigma | G0750-100G |
| Gelatin solution | Sigma | G1393-100ML |
| Glycine | Sigma | 50046-50 |
| Ham’s F12 | Invitrogen | 11765-054 |
| Hoescht 33342 | Molecular Probes | CAS 23491-52-3 |
| Human epidermal growth factor | Peprotech | AF-100-15 |
| Human fibroblast growth factor-basic | Peprotech | 100-18B |
| Iscove’s modified Dulbecco’s medium | Invitrogen | 12440-046 |
| L-Glutamine (200 mM) | Thermo Fisher | 25030-081 |
| Liberase | Roche | 05401151001 |
| Menadione | SigmaAldrich | M5750 |
| MES buffer (2-(N-Morpholino) ethanesulfonic acid | Sigma | M3671-50 |
| Oligomycin A | Abcam | CAS 1404-19-9 |
| Rotenone | Abcam | CAS 83-79-4 |
| RPMI | SigmaAldrich | R8758 |
| Sodium Pyruvate (100 mM) | Gibco | 11360070 |
| Thrombin | Roche | 10602400001 |
| TMRM (tetramethylehodamine methyl ester perchlorate | Thermo Fisher | T668 |
| TRI reagent | Sigma | T9424 |
| Trypsin-0.25% EDTA | Gibco | 25200-056 |
| Vectashield antifade mounting medium with DAPI | Vector Laboratories | H-1200 |
| Y-27632 dihydrochloride (ROCKi) | Tocris | 1254 |
| 2-mercaptoethanol | Sigma-Aldrich | M7522 |
| Critical Commercial Assays | | |
| CellDirect One-Step qRT–PCR kits | Invitrogen | 11753500 |
| CellROX Green | Invitrogen | C10444 |
| Click-iT Plus Terminal deoxynucleotidyl transferase (TdT) dUTP Nick-End Labeling (TUNEL) assay | Invitrogen | C10618 / C10246 |
| ExoSpin Columns | Cell Guidance Systems | EX01-8 |
| Human Cardiac Troponin 1 SimpleStep ELISA® Kit | Abcam | ab200016 |
| MitoSOX Red | Invitrogen | M36008 |
| Mouse L308 Array, Membrane | RayBiotech | AAM-BLM-1A-4 |
| ROS-Glo^TM^ H_2_O_2_ Assay | Promega | G8820 |
| Pierce 660nm protein assay reagent | Thermo Scientific | 22660 |
| Experimental Models: Cell Lines | | |
| Human dermal fibroblasts | Promocell | C-12302 |
| Human PSC-derived cardiomyocytes; IMR-90 | Sian Harding | <https://www.ncbi.nlm.nih.gov/pubmed/29086457> |
| Human PSC-derived cardiomyocytes: vCor.4U | Ncardia (Axiogenesis) | Ax-B-HC03-1M |
| Experimental Models: Organisms/Strains | | |
| C57BL/6 mice | Charles River | C57BL/6NCrl |
| Oligonucleotides | | |
| TaqMan Probe *CD29_ITGB1* | Thermo Fisher | Hs00559595_m1 |
| TaqMan Probe *CD44* | Thermo Fisher | Hs01075861_m1 |
| TaqMan Probe *CD73_NT5E* | Thermo Fisher | Hs00159686_m1 |
| TaqMan Probe *CD90_THY1* | Thermo Fisher | Hs00174816_m1 |
| TaqMan Probe *CD105* | Thermo Fisher | Hs00923996_m1 |
| TaqMan Probe *Gata4* | Thermo Fisher | Hs00171403_m1 |
| TaqMan Probe *Gata6* | Thermo Fisher | Hs00232018_m1 |
| TaqMan Probe *Hand1* | Thermo Fisher | Hs02330376_s1 |
| TaqMan Probe *Hand2* | Thermo Fisher | Hs00232769_m1 |
| TaqMan Probe *Isl1* | Thermo Fisher | Hs00158126_m1 |
| TaqMan Probe *Mb* | Thermo Fisher | Hs00193520_m1 |
| TaqMan Probe *Mef2a* | Thermo Fisher | Hs01050409_m1 |
| TaqMan Probe *Mef2c* | Thermo Fisher | Hs00231149_m1 |
| TaqMan Probe *Myh6* | Thermo Fisher | Hs01101425_m1 |
| TaqMan Probe *Myh7* | Thermo Fisher | Hs01110632_m1 |
| TaqMan Probe *Myh11* | Thermo Fisher | Hs00224610_m1 |
| TaqMan Probe *Myl2* | Thermo Fisher | Hs00166405_m1 |
| TaqMan Probe *Myl3* | Thermo Fisher | Hs00193520_m1 |
| TaqMan Probe *Myl7/Myl2a* | Thermo Fisher | Hs00221909_m1 |
| TaqMan Probe *Nppa* | Thermo Fisher | Hs00383230_g1 |
| TaqMan Probe *Nkx2.5* | Thermo Fisher | Hs00231763_m1 |
| TaqMan Probe *PDGFRa* | Thermo Fisher | Hs00998018_m1 |
| TaqMan Probe *Ptprc/CD45* | Thermo Fisher | Hs04189704_m1 |
| TaqMan Probe *Tbx5* | Thermo Fisher | Hs00361155_m1 |
| TaqMan Probe *Tbx20* | Thermo Fisher | Hs00396596_m1 |
| Software and Algorithms | | |
| Bioconductor package collection | Bioconductor project | <https://www.bioconductor.org> |
| CellOPTIQ | Clyde Biosciences | N/A |
| Fiji | ImageJ | https://imagej.net/Welcome |
| FlowJo Version 10 | FlowJo | N/A |
| Fluidigm software Version 4 | Fluidigm | N/A |
| HCS Studio BioApplication Cell Health Profiling V4 | Thermo Fisher | N/A |
| Prism Versions 6 and 7 | GraphPad Software Inc | <http://www.graphpad.com/scientific-software/prism/> |
| SeqMonk | Babraham Bioinformatics | <http://www.bioinformatics.babraham.ac.uk/projects/seqmonk/> |
| TrimGalore | Babraham Bioinformatics | <http://www.bioinformatics.babraham.ac.uk/projects/trim_galore/> |
| R software environment | The R Foundation | <https://cran.r-project.org> |
| Other | | |
| Amersham Hyperfilm ECL (18 x 24 cm) | GE Life Sciences | 28906836 |
| Array chips for 96 assays x 96 samples | Fluidigm | BMK-M10-96.96 |
| ArrayScan VTI High Content Screening platform | Cellomics | N/A |
| BioMark HD system | Fluidigm | N/A |
| CellOPTIQ | Clyde Biosciences | N/A |
| Cell strainer mesh 70 μm | BD Falcon | 352350 |
| Direct-zol RNA Miniprep kit | Zymo Research | T2051 |
| Half-area 96-well microclear black-bottom plates | Greiner | 675096 |
| HTS Transwell 96-well permeable supports with polyethylene terephthalate membranes and 1.0 micron pores | Corning | 3392 |
| RNeasy Mini kit | Qiagen | 74104 |
| 0.2 μm filter units FP30/0.2 CA-S | GE Healthcare | 10462200 |
| 96-MicroWell™ plate, flat bottomed wells, with lid | Nunclon, VWR | 734-2097 |
| Veriti Thermal Cycler | ABI, ThermoFisher | N/A |

**REFERENCES**

1 Noseda, M. *et al.* PDGFRalpha demarcates the cardiogenic clonogenic Sca1+ stem/progenitor cell in adult murine myocardium. *Nature communications* **6**, 6930, doi:10.1038/ncomms7930 (2015).
